# Ectodomain shedding of PLA2R1 is mediated by the metalloproteases ADAM10 and ADAM17

**DOI:** 10.1016/j.jbc.2024.107480

**Published:** 2024-06-17

**Authors:** Guillaume Dolla, Sarah Nicolas, Ligia Ramos dos Santos, Alexandre Bourgeois, Raphaëlle Pardossi-Piquard, Franck Bihl, Christelle Zaghrini, Joana Justino, Christine Payré, Pascal Mansuelle, Christoph Garbers, Pierre Ronco, Frédéric Checler, Gérard Lambeau, Agnès Petit-Paitel

**Affiliations:** 1 Centre National de la Recherche Scientifique, Inserm, Institut de Pharmacologie Moléculaire et Cellulaire, Sophia Antipolis, Université Côte d'Azur (UniCa), Valbonne, France; 2 Centre National de la Recherche Scientifique, Inserm, Institut de Pharmacologie Moléculaire et Cellulaire, Laboratoire d'Excellence DistALZ, Sophia Antipolis, Université Côte d'Azur (UniCa), Valbonne, France; 3 Plateforme de Protéomique de l'Institut de Microbiologie de la Méditerranée (IMM), Marseille Protéomique (MaP), Aix Marseille Université (AMU), Centre National de la Recherche Scientifique (CNRS) FR3479, Marseille, France; 4 Institute of Clinical Biochemistry, Hannover Medical School, Hannover, Germany; 5 Institut National de la Santé et de la Recherche Médicale (INSERM), UMR-S1155, Paris, France; 6 Sorbonne Université, Université Pierre et Marie Curie Paris 06, Paris, France

**Keywords:** PLA2R1, soluble PLA2R1, shedding, metalloproteases, ADAM10, ADAM17, membranous nephropathy, podocyte, inflammation

## Abstract

Phospholipase A2 receptor 1 (PLA2R1) is a 180-kDa transmembrane protein that plays a role in inflammation and cancer and is the major autoantigen in membranous nephropathy, a rare but severe autoimmune kidney disease. A soluble form of PLA2R1 has been detected in mouse and human serum. It is likely produced by proteolytic shedding of membrane-bound PLA2R1 but the mechanism is unknown. Here, we show that human PLA2R1 is cleaved by A Disintegrin And Metalloprotease 10 (ADAM10) and ADAM17 in HEK293 cells, mouse embryonic fibroblasts, and human podocytes. By combining site-directed mutagenesis and sequencing, we determined the exact cleavage site within the extracellular juxtamembrane stalk of human PLA2R1. Orthologs and paralogs of PLA2R1 are also shed. By using pharmacological inhibitors and genetic approaches with RNA interference and knock-out cellular models, we identified a major role of ADAM10 in the constitutive shedding of PLA2R1 and a dual role of ADAM10 and ADAM17 in the stimulated shedding. We did not observe evidence for cleavage by β- or γ-secretase, suggesting that PLA2R1 may not be a substrate for regulated intramembrane proteolysis. PLA2R1 shedding occurs constitutively and can be triggered by the calcium ionophore ionomycin, the protein kinase C activator PMA, cytokines, and lipopolysaccharides, *in vitro* and *in vivo*. Altogether, our results show that PLA2R1 is a novel substrate for ADAM10 and ADAM17, producing a soluble form that is increased in inflammatory conditions and likely exerts various functions in physiological and pathophysiological conditions including inflammation, cancer, and membranous nephropathy.

Phospholipase A2 receptor 1 (PLA2R1, M-type receptor) was discovered 30 years ago for its binding properties of secreted phospholipases A2 (sPLA2s) (1). PLA2R1 is a type I transmembrane N-glycosylated protein with a molecular mass of 180 kDa. It consists of a large N-terminal extracellular region with a cysteine-rich domain (CysR), a fibronectin-like type II domain and eight distinct C-type lectin-like domains (CTLDs), followed by a single transmembrane domain and a short C-terminal intracellular region (1). It belongs to the C-type lectin superfamily and has the macrophage mannose receptor (mannose receptor C-type 1, MRC1), Endo-180/UPARAP (mannose receptor C-type 2, MRC2), and DEC-205 (LY75) as closest paralogs (2).

PLA2R1 is a multiligand and multifunctional receptor (1, 3, 4). It binds many but not all exogenous (snake venoms) and endogenous (mammalian) sPLA2s. Upon binding, sPLA2s are inhibited, internalized, and degraded (3, 5, 6, 7, 8, 9, 10). PLA2R1 thus plays a role in controlling sPLA2 enzymatic activity in inflammatory conditions such as sepsis and asthma (3, 11, 12). PLA2R1 binds collagens I and IV, suggesting a role in cell adhesion, migration, and proliferation (6, 13, 14, 15). PLA2R1 has lectin properties and binds carbohydrate-conjugated proteins *in vitro*, yet its role as endogenous lectin is unclear (1, 7). PLA2R1 has also been proposed to function as a tumor-suppressor gene by inducing replicative or oncogene-induced cellular senescence through the p53 pathway and other signaling pathways (16, 17). Finally, over the last decade, PLA2R1 has attracted much attention in membranous nephropathy (MN), a rare but severe autoimmune kidney disease in which PLA2R1 was identified as the major autoantigen, with circulating anti-PLA2R1 autoantibodies present in a majority of MN patients (18, 19).

In 1995, while cloning human PLA2R1 (hPLA2R1) from the kidney, Ancian *et al.* found a particular form of soluble PLA2R1 which is encoded by an alternative mRNA splice variant and lacks the end of the CTLD8 domain, the transmembrane domain, and the intracellular domain (5). In 2000, Yokota *et al.* detected a higher level of soluble mouse PLA2R1 (mPLA2R1) in serum after administration of lipopolysaccharides (LPS) (20). As there was no evidence for an mRNA transcript directly coding for soluble mPLA2R1, the authors suggested that the mechanism leading to the production of this soluble form was different from the above human situation and would result from proteolytic shedding of membrane-bound mPLA2R1. In 2002, the same group provided evidence for a role of unknown metalloproteases (MPs) in the cleavage of mPLA2R1 (21). Finally, Watanabe *et al.* detected a soluble form of hPLA2R1 in the plasma of healthy donors but did not study the underlying mechanism of shedding (15). Soluble forms of PLA2R1 paralogs have also been described for MRC1 (22) and MRC2 (23).

Ectodomain shedding of transmembrane proteins concomitantly leads to the reduction of membrane-bound forms and the production of soluble forms with either new biological properties or antagonistic roles (24, 25, 26). Several members of the A Disintegrin and Metalloprotease (ADAM) family have emerged as major proteases controlling ectodomain shedding of membrane proteins in physiological and pathological conditions (25, 27, 28, 29, 30, 31). In this family, ADAM10 and ADAM17 have more than 100 substrates, such as receptors, cytokines, chemokines, and cell adhesion molecules. Examples include the Alzheimer disease–associated amyloid precursor protein (APP), tumor necrosis factor (TNF) and its receptors, Notch, and the interleukin-6 (IL-6) receptor. ADAM10 can be activated by intracellular Ca^2+^, as induced by ionomycin, whereas ADAM17 can be activated by various stimuli including phorbol esters (17, 25, 27, 29, 31, 32). Furthermore, ADAM10- and ADAM17-mediated proteolysis occurs as the first step of regulated intramembrane proteolysis (RIP), which is characterized by two consecutive protein cleavages near or within the plasma membrane (24, 33, 34). The membrane-bound protein is first cleaved in the ectodomain by either α-secretases such as ADAM10 or ADAM17 or β-secretases such as BACE1 to generate a soluble form of the protein. The remaining transmembrane C-terminal fragment then becomes a substrate for intramembrane cleavage by proteases including the Presenilin/γ-Secretase multiprotein complex (35). The protein fragments generated by these two cleavages are thus released extracellularly and intracellularly and are involved in diverse signaling pathways (24, 33, 34).

In this study, we identified hPLA2R1 as a novel substrate of ADAM10 and ADAM17, resulting in the production of a soluble form of hPLA2R1 and a membrane-bound C-terminal PLA2R1 fragment. The latter fragment did not undergo subsequent cleavage by either β- or γ-secretases, at least in our conditions. We identified the protease cleavage site within the extracellular juxtamembrane stalk of hPLA2R1 using complementary biochemical approaches. We observed a similar mechanism of shedding for orthologs and paralogs of hPLA2R1. We investigated the contribution of ADAM10 and ADAM17 in the shedding of hPLA2R1 using pharmacological and genetic approaches, in basal and stimulated conditions including inflammatory stimuli. Finally, we showed increased levels of soluble PLA2R1 in inflammatory conditions both *in vitro* and *in vivo*, in human podocytes and mice.

## Results

### Detection of cleavage fragments of hPLA2R1

**Figure 1:**
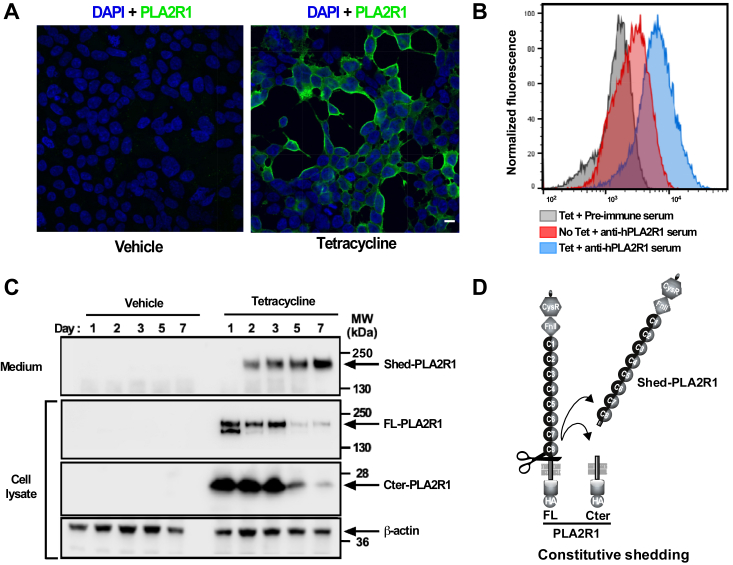
**hPLA2R1 is expressed at the plasma membrane and is constitutively shed in HEK293 cells.***A*, T-REx-293 cells stably expressing hPLA2R1 with a C-terminal HA tag were treated with vehicle (*left* panel) or 1 μg/ml tetracycline for 48 h. Cells were then fixed and labeled with anti-PLA2R1 antibodies targeting the extracellular region of hPLA2R1. Scale bar represents 10 μm. *B*, T-REx-293 cells stably expressing hPLA2R1 were treated with vehicle (*red* curve) or 1 μg/ml tetracycline (*gray* and *blue* curves) for 48 h and then analyzed by flow cytometry. Cell suspensions were labeled with rabbit pre-immune serum (*gray* curve) or anti-PLA2R1 serum (*blue* and *red* curves), then with a secondary anti-rabbit IgG-A488 antibody. *C*, T-REx-293 cells stably expressing hPLA2R1 were treated with vehicle (*left* lanes) or 1 μg/ml tetracycline (*right* lanes) for the indicated days. Protein samples from medium and cell lysate were analyzed by WB. The soluble form of hPLA2R1 (Shed-PLA2R1) was detected in medium with anti-PLA2R1 antibodies targeting the extracellular region of hPLA2R1 (*upper* panel). Full-length hPLA2R1 (FL-PLA2R1) and the small membrane-bound C-terminal fragment (Cter-PLA2R1) were detected in cell lysate with anti-HA antibodies (*middle* and *lower* panels). *D*, schematic representation of FL-PLA2R1 shedding, leading to the production of Shed-PLA2R1 and Cter-PLA2R1.

Although a soluble form of PLA2R1 has been described in mouse and human serum since several years (15, 21), the underlying mechanism of production has remained unknown. Because of the absence or low expression of endogenous hPLA2R1 in most human cell lines (https://www.proteinatlas.org/ENSG00000153246-PLA2R1/cell+line), we established T-REx-293 cells stably expressing hPLA2R1 (full-length membrane-bound form) with a C-terminal HA tag under the control of a tetracycline-inducible promoter. The tetracycline-dependent expression of hPLA2R1 at the cell surface was demonstrated by immunocytofluorescence (Fig. 1*A*) and flow cytometry (Fig. 1*B*). Upon tetracycline treatment, the cell surface expression of hPLA2R1 was strongly increased and a net shift in hPLA2R1 mean fluorescence intensity was observed when cells were labeled with anti-hPLA2R1 antibodies, as compared to vehicle-treated cells or tetracycline-treated cells labeled with pre-immune serum. Western blot (WB) analysis of cell lysate with anti-HA antibody revealed a robust expression of full-length hPLA2R1 (hereafter called FL-PLA2R1 when specifically referring to the full-length protein) 1 to 3 days after induction by tetracycline (Fig. 1*C*, middle panel). At day 1, two bands of equal intensity were observed at approximately 170 and 180 kDa, possibly corresponding to different forms of N-glycosylated hPLA2R1 (5). At days 2 and 3, only the upper band of hPLA2R1 was observed, while expression was reduced at days 5 and 7 (Fig. 1*C*, middle panel). For the experiments described below, we thus chose to treat cells for 2 days with tetracycline for optimal expression of hPLA2R1.

To search for cleavage fragments of hPLA2R1, we analyzed protein samples from medium and cell lysate of hPLA2R1-expressing T-REx-293 cells obtained at different time points after addition of tetracycline (Fig. 1*C*). By WB and using a specific antibody targeting the extracellular region of hPLA2R1, we detected no signal at any time point in the culture medium of vehicle-treated cells, but a major band with a molecular mass of about 170 kDa in the medium of cells induced by tetracycline, indicating the release of a soluble form of hPLA2R1, hereafter called Shed-PLA2R1 (Fig. 1*C*, upper panel). Shed-PLA2R1 was detected at all time points after addition of tetracycline, showing a constitutive production over the time course of induction. Shed-PLA2R1 was still detected in the culture medium after high-speed ultracentrifugation, indicating that it is not due to membrane-bound PLA2R1 from cell debris or extracellular vesicles secreted from cells (Fig. S1*A*). By WB and using the anti-HA antibody specifically targeting the C-terminal HA tag added to the cytoplasmic domain of hPLA2R1, we detected a band of approximately 10 kDa in the cell lysate of hPLA2R1-expressing T-REx-293 cells treated with tetracycline, whereas no signal was detected in vehicle-treated cells (Fig. 1*C*, lower panel). This band corresponds to the C-terminal membrane-bound PLA2R1 fragment (hereafter called Cter-PLA2R1) and is likely the counterpart of Shed-PLA2R1. Interestingly, Shed-PLA2R1 was found to accumulate over the time course of the experiment, at the expense of FL-PLA2R1, showing the constitutive conversion of membrane-bound hPLA2R1 into a soluble form that remains stable over time. On the other hand, Cter-PLA2R1 accumulates up to day 3 and then disappears over the time course of the experiment. This suggests distinct fates for Shed-PLA2R1 and Cter-PLA2R1 once produced, with instability of Cter-PLA2R1 in cellular membranes.

Altogether, these results suggest a mechanism of constitutive ectodomain shedding of hPLA2R1 by unknown proteases when expressed in hPLA2R1-expressing T-REx-293 cells. These proteases cleave full-length PLA2R1 (FL-PLA2R1, 180 kDa) to release in cell medium the large extracellular region of hPLA2R1 as a soluble form (Shed-PLA2R1, 170 kDa) and to leave in the cell membrane the small C-terminal counterpart (Cter-PLA2R1, 10 kDa) that is eventually cleared off over time (Fig. 1*D*).

### Shedding of PLA2R1 occurs within the juxtamembrane stalk

**Figure 2:**
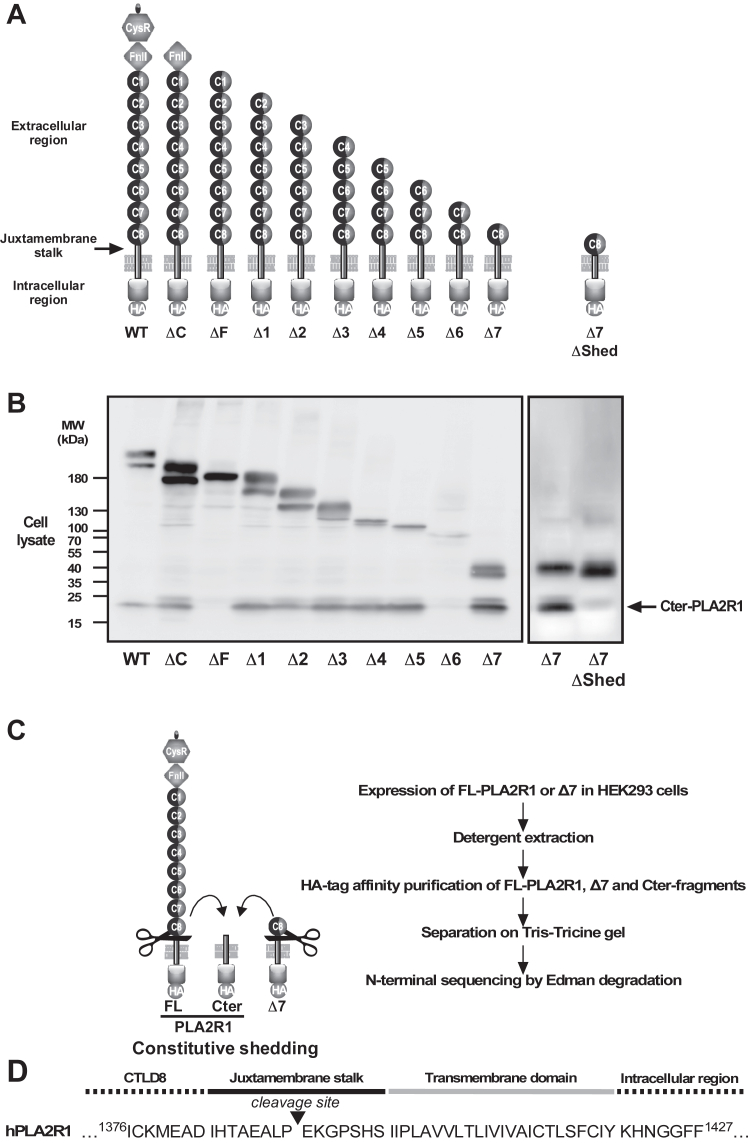
**Structural determinants of hPLA2R1 cleavage in HEK293 cells.***A*, schematic representation of serial deletion mutants of the hPLA2R1 extracellular region that were transfected into HEK293 cells. The different domains of hPLA2R1 are indicated: CysR: cysteine-rich domain, FnII: fibronectin type II domain, C1 to C8: CTLD1 to CTLD8 domains, the short juxtamembrane stalk, and the transmembrane domain (see panel *D*), and the intracellular region with the C-terminal HA tag. The Δ7ΔShed mutant corresponds to the Δ7 mutant in which the juxtamembrane stalk is deleted. *B*, cell lysate from HEK293 cells transfected with the deletion mutants were analyzed by WB with anti-HA antibody to detect the mutants and the Cter-PLA2R1 fragment. *C*, strategy of purification and sequence identification of the C-terminal fragments generated from FL-PLA2R1 and Δ7 expressed in HEK293 cells (cells were not stimulated before purification). *D*, N-terminal Edman sequencing identified the same ^1391^EKGPSHSIIP^1400^ sequence for the purified C-terminal fragments from FL-PLA2R1 and Δ7. The sequence is shown in the context of the juxtamembrane stalk of hPLA2R1, from Ile1383 to Ser1397.

To identify the structural determinants of hPLA2R1 cleavage, we generated a series of HA-tagged deletion mutants of hPLA2R1 in which the extracellular domains were removed one by one, from the N-terminal CysR domain to the C-terminal CTLD8 domain (Fig. 2*A*). WB analysis of cell lysate from transfected HEK293 cells indicated that the mutants are shed, as shown by the detection of Cter-PLA2R1 (Fig. 2*B*). The Δ7 mutant, which is the shortest deletion mutant, contains the CTLD8 domain, the juxtamembrane stalk, the transmembrane domain, and the C-terminal intracellular region of hPLA2R1 (Fig. 2*A*). It was well expressed and efficiently shed (Fig. 2*B*). The ΔF mutant, which begins at the CTLD1 domain, was not cleaved in our experimental conditions, while data for the Δ6 mutant were inconclusive, because of weak expression. Overall, the results suggest that the different extracellular domains of hPLA2R1 are dispensable for proteolytic cleavage and that the essential information for cleavage resides near the plasma membrane, likely within the juxtamembrane stalk located between the CTLD8 domain and the plasma membrane (Fig. 2*A*).

To identify more precisely the structural elements involved in PLA2R1 shedding, we generated a mutant called Δ7ΔShed, corresponding to Δ7 in which the juxtamembrane stalk of 15 amino acids is deleted (Fig. 2*A*). WB analysis showed that, unlike Δ7, there was no detection of Cter-PLA2R1 in the cell lysate of HEK293 cells transfected with Δ7ΔShed (Fig. 2*B*). Similar data were obtained for the ΔShed mutant of hPLA2R1, where the juxtamembrane stalk was deleted (Fig. S1*B*). Accordingly, analysis of Shed-PLA2R1 in cell medium from WT *versus* ΔShed hPLA2R1 and of Shed-Δ7 from Δ7 *versus* Δ7ΔShed mutants showed much reduced amounts for the ΔShed mutants (Fig. S1*B*). These results indicate that the short juxtamembrane stalk (15 amino acids) of hPLA2R1 is essential for shedding.

To determine the exact proteolytic cleavage site, we transfected HEK293 cells with PLA2R1 or Δ7 with a C-terminal HA tag and purified the C-terminal PLA2R1 fragments resulting from constitutive shedding. The proteins were separated on Tris-Tricine gels, electroblotted, and submitted to N-terminal sequencing by Edman degradation (Fig. 2*D*). The Cter-PLA2R1 fragments from the two constructs had identical molecular masses, and N-terminal sequencing provided similar sequencing data. In both cases, the cleavage of hPLA2R1 occurred within the 15 amino acid juxtamembrane stalk, after proline 1390 (Fig. 2*D*). To confirm this result, we generated a set of point and short deletion mutants within the juxtamembrane stalk in Δ7 (Fig. S1*C*). Deletion of the six amino acids at the cleavage site (Δ1388-1393) fully abolished cleavage, whereas shorter deletions (Δ1391-1393, Δ1388-1390) or point mutations at proline 1390 by acidic or aliphatic amino acids (P1390D, P1390G, P1390A) had no or limited effect on the production of Cter-PLA2R1 (Fig. S1*C*).

### Orthologs and paralogs of hPLA2R1 are constitutively shed in HEK293 cells

**Figure 3:**
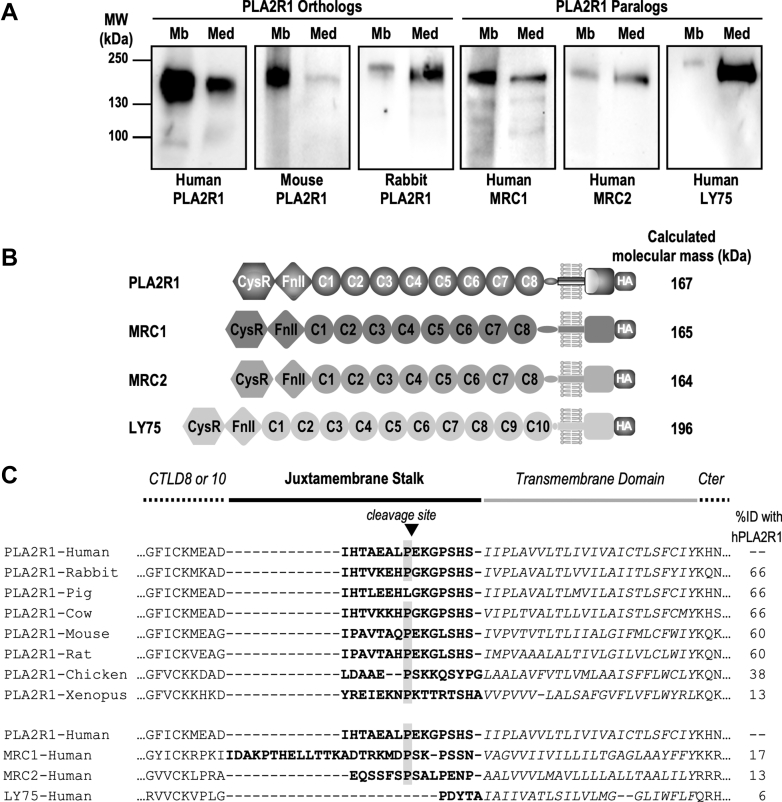
**Orthologs and paralogs of hPLA2R1 are shed in HEK293 cells.***A*, human, rabbit, and mouse PLA2R1 orthologs as well as human MRC1, MRC2, and LY75 paralogs were transiently transfected in HEK293 cells. Protein samples from cell lysate (Mb) and medium (Med) were analyzed by WB with specific antibodies under reducing or nonreducing conditions, as specified in the experimental procedures. Different sample volumes were loaded, depending on expression level and reactivity of antibodies. The soluble forms of mouse PLA2R1, human MRC1, and human LY75 were precipitated with TCA from cell medium. *B*, schematic representation of C-terminally HA-tagged constructs coding for the full-length human paralogs: MRC1 (8 CTLDs), MRC2 (8 CTLDs), and LY75 (10 CTLDs). *C*, amino-acid sequence and percentage of identity of the PLA2R1 juxtamembrane stalk among orthologs and paralogs. The proline residue at which the cleavage of hPLA2R1 occurs is well-conserved among orthologs and paralogs. Alignment was performed with muscle. Note the different lengths of the juxtamembrane stalk in hPLA2R1 paralogs, as also illustrated in panel *B*. TCA, trichloroacetic acid.

We then tested whether orthologs and paralogs of hPLA2R1 also undergo ectodomain shedding. We transfected HEK293 cells with expression plasmids coding for human, rabbit, and mouse PLA2R1 orthologs and for human MRC1, MRC2, and LY75 paralogs (Fig. 3, *A* and *B*). WB analysis shows the presence of a soluble form in the cell medium for each ortholog and paralog, which has a slightly lower molecular mass, as compared to the membrane-bound form observed in cell lysate (Fig. 3*A*). Interestingly, the juxtamembrane stalk is fairly well conserved between orthologs and paralogs of hPLA2R1 (yet of different lengths among paralogs) and includes in most cases a proline residue at the cleavage site identified for hPLA2R1 (Fig. 3*C*).

### Shedding of hPLA2R1 is stimulated by PMA and ionomycin

**Figure 4:**
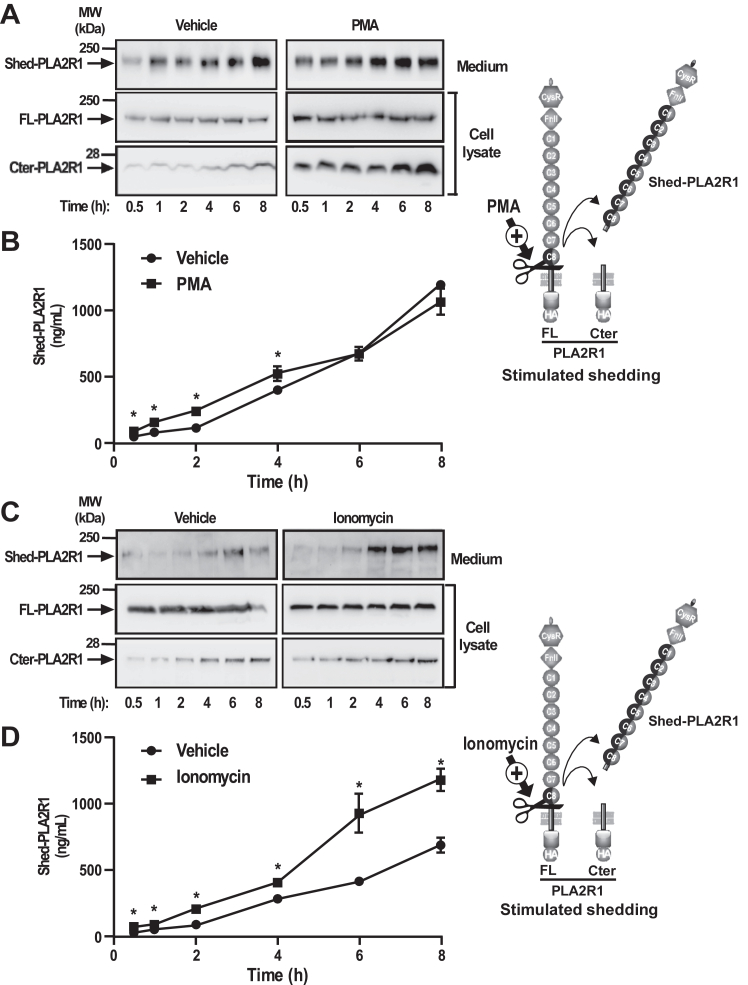
**PMA and ionomycin increase the shedding of hPLA2R1 in HEK293 cells.***A*, hPLA2R1 expression was induced in T-Rex-293 cells by tetracycline treatment (1 μg/ml) for 48 h. Medium was changed and cells were treated with vehicle or the phorbol ester PMA (200 nM) for the indicated time. Immunoblots of medium and cell lysate were probed with anti-PLA2R1 antibodies targeting the extracellular region of hPLA2R1 to detect Shed-PLA2R1 or anti-HA antibodies to detect FL-PLA2R1 and Cter-PLA2R1. *B*, protein samples from medium were analyzed by time-resolved fluoroimmunoassay (TRFIA) to detect Shed-PLA2R1 (n = 3, ∗*p* value < 0.05). *C* and *D*, T-Rex-293 cells were treated as above but with ionomycin (2.5 μM) for the indicated time. Both PMA and ionomycin stimulate the shedding of hPLA2R1, but with different time courses.

The MPs involved in ectodomain shedding of membrane proteins are often activated by protein kinase C (PKC) and intracellular Ca^2+^ (36). Therefore, we examined whether phorbol 12-myristate 13-acetate (PMA), a PKC activator, and ionomycin, a Ca^2+^ ionophore, could increase the shedding of hPLA2R1 in T-REx-293 cells. Treatment with PMA (Fig. 4, *A* and *B*) and ionomycin (Fig. 4, *C* and *D*) increased the shedding of hPLA2R1 in a time-dependent manner, as shown by increased levels of Shed-PLA2R1 and Cter-PLA2R1 observed by WB (Fig. 4, *A* and *C*). This observation was confirmed more quantitatively using a time-resolved fluoroimmunoassay (TRFIA, see experimental procedures and Fig. S2) to specifically detect Shed-PLA2R1 in cell medium (Fig. 4, *B* and *D*). The effect of PMA on shedding was observed during the first 4 h of treatment, after which it was no longer significant (Fig. 4, *A* and *B*). The effect of ionomycin was more robust and observed throughout the time course of the experiment (Fig. 4, *C* and *D*). Accordingly, levels of Cter-PLA2R1 in cell lysate were increased by PMA and ionomycin (Fig. 4, *A* and *C*). Altogether, these results indicate that the shedding of hPLA2R1 occurs in both basal and stimulated conditions.

### Shedding of hPLA2R1 is mediated by MPs belonging to the ADAM family

**Figure 5:**
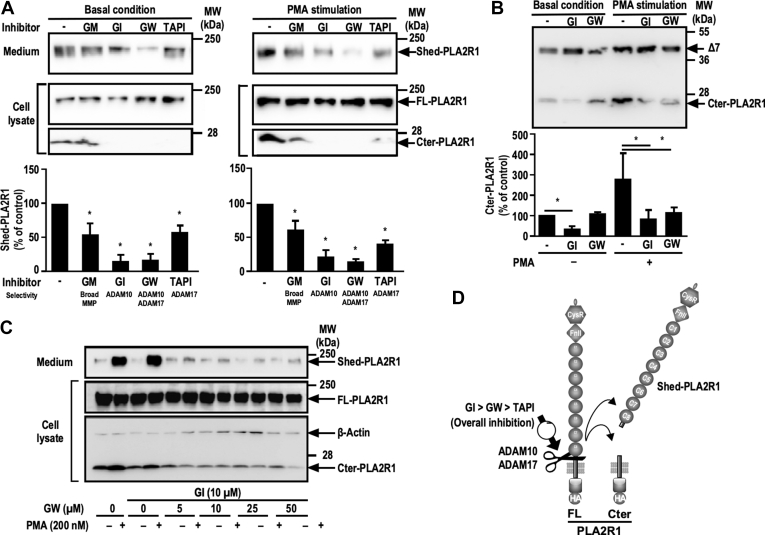
**Pharmacological inhibitors of ADAM10 and ADAM17 block the shedding of hPLA2R1 in HEK293 cells.***A*, hPLA2R1 expression was induced in T-Rex-293 cells by tetracycline treatment (1 μg/ml) for 48 h. Medium was changed and cells were pretreated for 30 min with the general metalloprotease inhibitor GM6001 (GM, 10 μM), the preferential ADAM10 inhibitor GI254023X (GI, 10 μM), the ADAM10/17 dual inhibitor GW280264X (GW, 10 μM), or the preferential ADAM17 inhibitor TAPI-1 (TAPI, 10 μM), and then vehicle or PMA (200 nM) was added for 1 h. Histograms show the quantification of Shed-PLA2R1 in cell medium from three independent WB experiments and is expressed as the % of Shed-PLA2R1 from tetracycline-induced vehicle-treated cells (n = 3, ∗*p* value < 0.05). Immunoblots of medium (*upper* panels) and cell lysate (*lower* panels) were probed with anti-PLA2R1 antibodies targeting the extracellular region of hPLA2R1 to detect Shed-PLA2R1 or anti-HA antibodies to detect FL-PLA2R1 and Cter-PLA2R1. *B*, HEK293 cells transfected with Δ7 were pretreated for 30 min with GI or GW (10 μM) and then vehicle or PMA (200 nM) was added for 1 h. Protein samples from cell lysate were analyzed by WB with anti-HA antibodies to detect the full-length form of Δ7 (about 40 kDa) and its C-terminal fragment which is identical to Cter-PLA2R1, as shown in Figure 2. Histograms show the quantification of Cter-PLA2R1 in cell lysate from three independent WB experiments and is expressed as the % of Cter-PLA2R1 from tetracycline-induced vehicle-treated cells (n = 3, ∗*p* value < 0.05). *C*, hPLA2R1 expression was induced in T-Rex-293 cells by tetracycline treatment (1 μg/ml) for 48 h. Medium was changed and cells were pretreated for 30 min with vehicle or GI (10 μM) and the indicated concentrations of GW, then vehicle or PMA (200 nM) was added for 1 h. Immunoblots of medium (*upper* panels) and cell lysate (*lower* panels) were probed with anti-PLA2R1 antibodies targeting the extracellular region of hPLA2R1 to detect Shed-PLA2R1 or anti-HA antibodies to detect FL-PLA2R1 and Cter-PLA2R1. β-actin served as a loading control. *D*, schematic representation summarizing the respective effects of GI (ADAM10 specific inhibitor), GW (ADAM10/17 dual inhibitor), and TAPI (ADAM17 inhibitor) in the shedding of hPLA2R1.

To identify the MPs involved in the shedding of hPLA2R1, we tested the effect of various pharmacological inhibitors under basal and stimulated conditions. The inhibitors included the broadly-specific matrix metalloprotease inhibitor GM6001 (GM), the preferential ADAM10 inhibitor GI254023X (GI), the ADAM10/ADAM17 dual inhibitor GW280264X (GW), and the TACE/ADAM17 (TNF-α converting enzyme/ADAM17) inhibitor TAPI-1 (28, 37). As shown by WB, in basal *versus* PMA-stimulated conditions, the different inhibitors reduced to various extents the amount of Shed-PLA2R1 in cell medium and that of Cter-PLA2R1 in cell lysate (Fig. 5*A*). GI and GW were the most potent inhibitors in both basal and PMA-stimulated conditions, suggesting a role of both ADAM10 and ADAM17. We then tested the effect of GI and GW on HEK293 cells expressing Δ7 (Fig. 5*B*). In basal conditions, GI but not GW reduced the amount of Cter-PLA2R1, suggesting a central role of ADAM10. However, in PMA-stimulated conditions, the two inhibitors had similar inhibitory effects, suggesting a role of both ADAM10 and ADAM17 (Fig. 5*B*).

We also tested the combined effect of GI and GW on PMA-regulated shedding of hPLA2R1 (Fig. 5*C*). T-REx-293 cells expressing hPLA2R1 were pretreated for 30 min with vehicle or GI in the absence or presence of various concentrations of GW and then treated with PMA for 1 h to stimulate shedding. Medium and cell lysate were analyzed by WB to detect Shed-PLA2R1, FL-PLA2R1, and Cter-PLA2R1. The results confirmed the effect of PMA on hPLA2R1 shedding, as evidenced by increased levels of Shed-PLA2R1 in medium and Cter-PLA2R1 in cell lysate (Fig. 5*C*, upper and lower panels). In the presence of PMA, the levels of Shed-PLA2R1 and Cter-PLA2R1 were not affected by GI. However, addition of GW over GI almost fully blocked the PMA-induced levels of Shed-PLA2R1 and Cter-PLA2R1, suggesting a role of ADAM17 in PMA-stimulated conditions (Fig. 5*C*).

Overall, the results from this pharmacological approach indicate that GI is usually the most potent inhibitor, followed by GW and TAPI-1, yet this potency depends on basal *versus* PMA-stimulated conditions (Fig. 5*D*). This in turn suggests that ADAM10 (specifically inhibited by GI) is mostly responsible for the constitutive shedding of hPLA2R1 in basal conditions, while both ADAM10 and ADAM17 (inhibited by GW) contribute to the shedding of hPLA2R1 when cells are stimulated with PMA.

### ADAM10 plays a central role in PLA2R1 shedding and ADAM17 contributes in stimulated conditions

**Figure 6:**
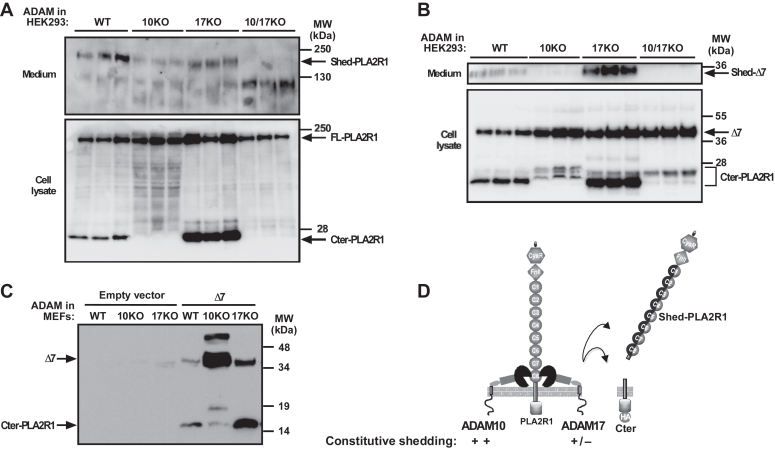
**Genetic deletion shows a prominent role of ADAM10 in the constitutive shedding of hPLA2R1 in HEK293 cells and MEFs.***A*, HEK293 cells either WT or deficient (*via* the CRISPR/Cas9-system) for ADAM10 (10KO), ADAM17 (17KO), or both proteases (10/17KO) were transfected with hPLA2R1-HA for 48 h. After transfection, medium was changed and cells were incubated for 2 h without stimulation. Protein samples from medium and cell lysate (in triplicates) were analyzed by WB with anti-PLA2R1 antibodies targeting the extracellular region of hPLA2R1 to detect Shed-PLA2R1 (medium) or anti-HA antibodies to detect FL-PLA2R1 and Cter-PLA2R1 (cell lysate). *B*, same as above but for transfection of the Δ7 mutant. Protein samples from medium and cell lysate were analyzed by WB with anti-Flag antibodies to detect Shed-Δ7 (*upper* panel) or anti-HA antibodies to detect full-length Δ7 and Cter-PLA2R1 (*lower* panel), respectively. *C*, mouse embryonic fibroblasts (MEFs) either WT or KO for ADAM10 (10KO) or ADAM17 (17KO) were transfected with Δ7 for 48 h. Protein samples from cell lysate were analyzed by WB with anti-HA antibodies to detect full-length Δ7 and Cter-PLA2R1, respectively. *D*, schematic representation showing the prominent role of ADAM10 over ADAM17 in the constitutive shedding of hPLA2R1.

To validate the above conclusions drawn from the pharmacological approach, we analyzed the shedding of hPLA2R1 and Δ7 in three different cellular models deficient for either ADAM10, ADAM17, or both enzymes at the gene level. First, we used siRNAs to reduce the expression of ADAM10 or ADAM17 and examined the effect on shedding of hPLA2R1 (Fig. S3). We transfected hPLA2R1-expressing T-REx-293 cells with siRNA specifically targeting ADAM10 or ADAM17 *versus* scrambled siRNA. As shown by RT-qPCR, siRNA silencing was more effective for ADAM10 than for ADAM17 (Fig. S3*A*). In basal conditions, WB analysis of medium and cell lysate showed that silencing of endogenous ADAM10 partially reduced the levels of Shed-PLA2R1 and Cter-PLA2R1, whereas silencing of ADAM17 had little effect (Fig. S3*B*). In PMA-stimulated conditions, silencing of either ADAM10 or ADAM17 partially reduced the levels of Shed-PLA2R1 and Cter-PLA2R1 (Fig. S3*B*). Second, we employed genetically-modified HEK293 cells fully deficient for ADAM10, ADAM17, or both proteases, generated using the CRISPR/Cas9-system (38) (Fig. 6, *A* and *B*). Deletion of ADAM10 and ADAM17 at the protein level was validated by WB (Fig. S4*A*). In basal conditions, a strong reduction of shedding was observed in hPLA2R1-transfected cells deficient for ADAM10 (ADAM10 KO) or both proteases (ADAM10/ADAM17 KO), as shown by WB analysis measuring Shed-PLA2R1 and Cter-PLA2R1 in medium and cell lysate, respectively (Fig. 6*A*). Conversely, shedding remained high in cells deficient for ADAM17, with efficient release of Shed-PLA2R1 and Cter-PLA2R1 (Fig. 6*A*). We also transfected HEK293 cells deficient for ADAM10, ADAM17, or both proteases with the Δ7 mutant and analyzed shedding by WB (Fig. 6*B*). In basal conditions, deficiency in ADAM10 in both single and double KO cells led to the disappearance of the prototypical Cter-PLA2R1 but interestingly revealed the appearance of new Cter-PLA2R1 fragments of slightly higher molecular masses (Fig. 6*B*, lower panel). Conversely, shedding of Δ7 remained efficient in ADAM17 KO cells (Fig. 6*B*). Third, we used mouse embryonic fibroblasts (MEFs) deficient for either ADAM10 or ADAM17 transfected with Δ7 (39). The data confirmed that deficiency in ADAM10 led to a strong decrease in the production of Cter-PLA2R1 in basal conditions (Fig. 6*C*). Conversely, deficiency in ADAM17 did not affect the production of Cter-PLA2R1 (Fig. 6*C*).

**Figure 7:**
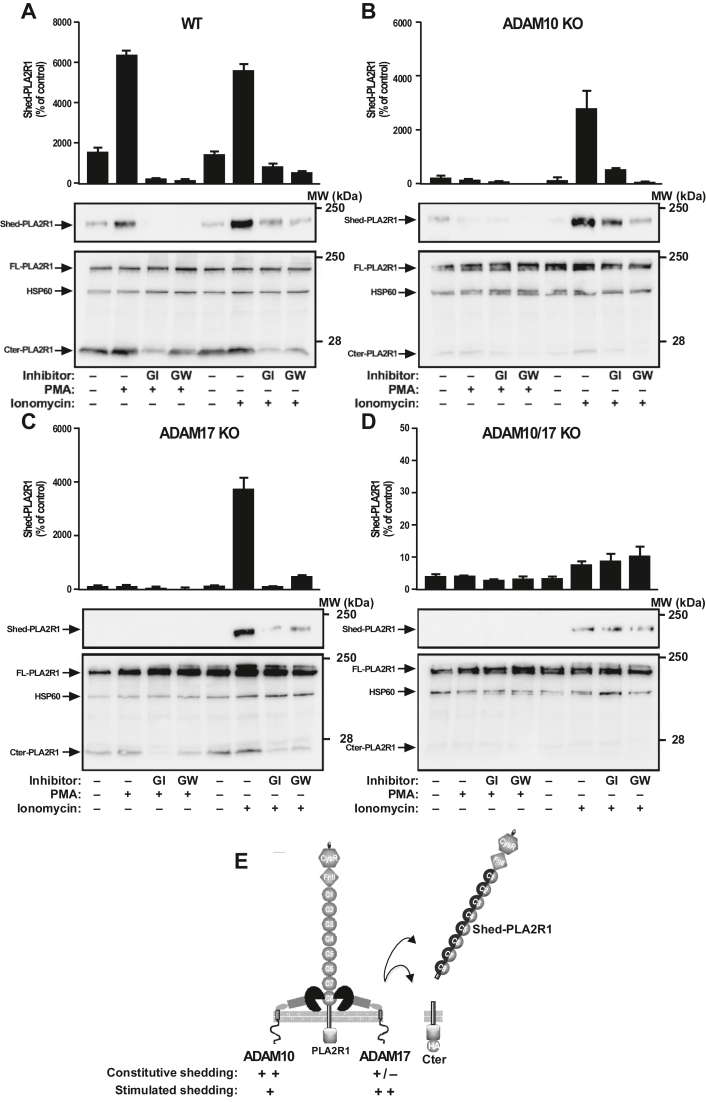
**Genetic deletion shows that both ADAM10 and ADAM17 contribute to the stimulated shedding of hPLA2R1 in HEK293 cells.***A*–*D*, HEK293 cells, either WT or KO for ADAM10 (10KO), ADAM17 (17KO), or both proteases (10/17KO), were transfected with a plasmid coding for hPLA2R1-HA for 48 h. After transfection, medium was changed, cells were pretreated for 30 min with the indicated ADAM inhibitor (10 μM), then vehicle, PMA (200 nM), or ionomycin (2.5 μM) was added for 2 h. Protein samples from medium were analyzed by time-resolved fluoroimmunoassay (TRFIA) to quantify Shed-PLA2R1 (n = 3). Please note the change in scale for ADAM10/17 KO cells *versus* other cell lines. Protein samples from medium and cell lysate were analyzed by WB with anti-PLA2R1 antibodies targeting the extracellular region of hPLA2R1 to detect Shed-PLA2R1 (*upper* panels) or anti-HA antibodies to detect FL-PLA2R1 and Cter-PLA2R1 (*lower* panels). HSP60 was used as a loading control. *E*, schematic representation showing the respective role of ADAM10 over ADAM17 in the constitutive *versus* stimulated shedding of hPLA2R1.

We also determined the respective role of ADAM10 and ADAM17 in the stimulated shedding of hPLA2R1 triggered by PMA or ionomycin in HEK293 cells WT or deficient for ADAM10, ADAM17, or both proteases, and in the presence of GI and GW. In WT cells, we observed a strong stimulating effect of PMA and ionomycin in the production of Shed-PLA2R1, as analyzed by TRFIA and WB, and of Cter-PLA2R1 by WB (Fig. 7*A*). These stimulating effects were completely inhibited by pretreatment with GI or GW (Fig. 7*A*). In contrast, in ADAM10 or ADAM17 KO cells, shedding of PLA2R1 was no longer stimulated by PMA but still activatable by ionomycin (Fig. 7, *B* and *C*). In cells with genetic deletion of both proteases, a sharp reduction of shedding activity was observed, illustrated in particular by the very low levels of Shed-PLA2R1 measured by TRFIA, in both basal and stimulated conditions. Interestingly, shedding of hPLA2R1 was still observed in either basal conditions or after stimulation by ionomycin, but it occurred at a much lower rate and was insensitive to GI and GW inhibitors (Fig. 7*D*). These observations are in line with the presence of different but faint Cter-PLA2R1 fragments observed in the HEK293 double KO cells transfected with Δ7 (Fig. 6*B*).

Altogether, we conclude that ADAM10 plays a central role in the shedding of PLA2R1 in both basal and stimulated conditions, while ADAM17 also contributes to shedding and more efficiently in stimulated conditions. Furthermore, analysis of the shedding activity in HEK293 cells deficient for both ADAM10 and ADAM17 revealed the presence of at least a third yet unknown protease capable of shedding hPLA2R1, which could cleave the receptor at a much lower rate, could be stimulated by ionomycin and was insensitive to GI and GW inhibitors.

### hPLA2R1 is not a substrate for β-secretase and does not undergo RIP

**Figure 8:**
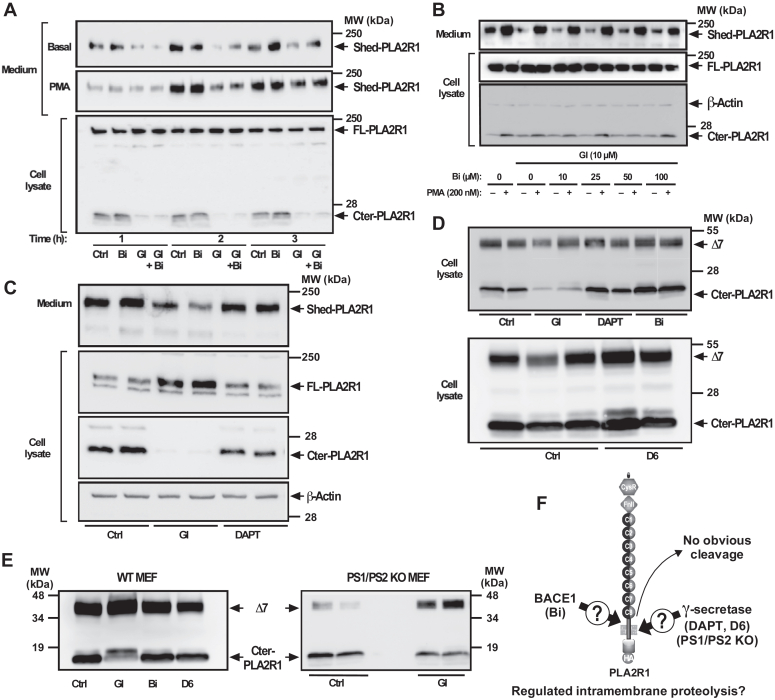
**hPLA2R1 does not appear as a substrate for β-secretase and γ-secretase in HEK293 cells and MEFs.***A–C*, hPLA2R1 expression was induced in T-Rex-293 cells by tetracycline treatment (1 μg/ml) for 48 h. *A*, medium was changed and cells were pretreated for 30 min with the β-secretase BACE-1 inhibitor (Bi, 100 μM), the ADAM10 inhibitor GI254023X (GI, 10 μM) or both, then vehicle or PMA (200 nM) was added for 1, 2, or 3 additional hours. *Upper* panels, WB of medium from unstimulated and PMA-stimulated cells probed with anti-PLA2R1 antibodies targeting the extracellular region of hPLA2R1 to detect Shed-PLA2R1. *Lower* panel, WB of cell lysate with anti-HA antibodies to detect FL-PLA2R1 and Cter-PLA2R1. *B*, medium was changed and cells were pretreated with GI (10 μM) and/or the indicated concentrations of Bi for 30 min, then vehicle or PMA (200 nM) was added for 1 h. Immunoblots of medium (*upper* panel) and cell lysate (*lower* panels) were probed with anti-PLA2R1 antibodies targeting the extracellular region of hPLA2R1 to detect Shed-PLA2R1 or anti-HA antibodies to detect FL-PLA2R1 and Cter-PLA2R1. Actin was used as a loading control. *C*, medium was changed and cells were pretreated with GI (30 μM) or the γ-secretase inhibitor DAPT (10 μM) for 1 h. Immunoblots of medium (*upper* panel) and cell lysate (*lower* panels) were probed with anti-PLA2R1 antibodies targeting the extracellular region of hPLA2R1 to detect Shed-PLA2R1 or anti-HA antibodies to detect FL-PLA2R1 and Cter-PLA2R1. Actin was used as a loading control. *D*, HEK293 cells were transfected with Δ7 and treated for 1 h with vehicle (Ctrl), GI (30 μM), DAPT (10 μM), or Bi (30 μM) (*upper* panel) and with vehicle or D6 inhibitor (12.5 μM) (*lower* panel). Protein samples from cell lysate were analyzed by WB with anti-HA antibodies to detect the full-length form of Δ7 and Cter-PLA2R1. *E*, mouse embryonic fibroblasts (MEFs), either WT or KO for presenilins (PS1/PS2 double KO) were transfected with Δ7 for 48 h. Protein samples from cell lysate were analyzed by WB with anti-HA antibodies to detect full-length Δ7 and Cter-PLA2R1, respectively. WT MEFs were also transfected with Δ7 and treated with the above inhibitors. *F*, schematic representation showing that hPLA2R1 is not cleaved by β-secretase and γ-secretase in HEK293 cells and MEFs in our settings.

In the processing of APP, two types of soluble forms are generated: the first by the action of an α-secretase activity, which is mediated by one or more ADAM proteases (ADAM9, 10, 17, and 19 being the most likely candidates); the second by the action of a β-secretase activity, namely the β-site APP-cleaving enzymes (BACE1 and BACE2) (24). We thus asked whether hPLA2R1 could behave as a substrate for BACEs. We induced the expression of hPLA2R1 in T-REx-293 cells with tetracycline for 48 h, then treated cells with either the ADAM10 inhibitor GI or the BACE1 inhibitor (Bi) for 30 min, and finally with or without PMA for 1 to 3 h. Unlike GI, Bi did not inhibit the production of Shed-PLA2R1 in cell medium neither in basal nor in PMA-stimulated conditions (Fig. 8*A*). Bi also did not affect the levels of Cter-PLA2R1, even when T-REx-293 cells expressing hPLA2R1 were treated for up to 6 h (Figs. 8*A* and S5). To analyze the effect of combined inhibition of α- and β-secretase activity on hPLA2R1 cleavage, we treated cells with a fixed concentration of GI and increasing concentrations of Bi, in basal and PMA-stimulated conditions. Here again, Bi treatment had no effect on the production of Shed-PLA2R1 in medium and of Cter-PLA2R1 in cell lysate (Fig. 8*B*). Altogether, the results suggest that hPLA2R1 is not cleaved by a β-secretase-like activity, at least in our HEK293 cells and experimental conditions.

After release of the ectodomains by a first cleavage event, several but not all type I transmembrane proteins can be cleaved secondarily by a presenilin/γ-secretase activity within the transmembrane domain, to release cytosolic fragments that move to the nucleus and induce gene transcription (24, 33, 34). This mechanism, called RIP, influences processes as diverse as cellular differentiation, lipid metabolism, and the response to unfolded proteins. We asked whether a RIP mechanism may occur in hPLA2R1. To test the possibility of cleavage by a presenilin/γ-secretase-like activity, we examined the effect of DAPT, a γ-secretase inhibitor. T-REx-293 cells were treated with tetracycline to induce hPLA2R1 expression, then treated for 1 h with vehicle, GI254023X, or DAPT. In basal conditions, DAPT treatment had no effect on the release of Shed-PLA2R1 in the cell medium and of Cter-PLA2R1 in cell lysate (Fig. 8*C*).

The absence of hPLA2R1 cleavage by β- and γ-secretase activities was confirmed by analyzing the processing of Δ7, which is expressed at higher levels than hPLA2R1 in HEK293 cells and is efficiently cleaved by ADAM10 and ADAM17 to release Cter-PLA2R1 (Fig. 2). In basal conditions, treatment with GI254023X greatly reduced the levels of Cter-PLA2R1, whereas neither Bi nor DAPT had an effect (Fig. 8*D*). Shedding of Δ7 was also insensitive to D6, another potent γ-secretase inhibitor (Fig. 8*D*). Finally, Δ7 was transfected in MEFs, either WT or knock-out for presenilins 1 et 2 (PS1/PS2), leading to the absence of γ-secretase activity (39). The lack of expression of PS1 and PS2 was validated by WB (Fig. S4*C*). In both types of MEFs, Δ7 was expressed as a membrane protein of about 40 kDa in cell lysate, and the production of the corresponding Cter-PLA2R1 fragment was unchanged, indicating that the lack of presenilins did not modify the features of Δ7 cleavage (Fig. 8*E*). In agreement with the results obtained in HEK293 cells, Δ7 cleavage in MEFs was insensitive to the β- and γ-secretase inhibitors Bi and D6 but was sensitive to the ADAM10 inhibitor GI254023X (Fig. 8*E*).

In conclusion, in our settings, we did not observe evidence for an obvious cleavage of hPLA2R1 by β- and γ-secretases (Fig. 8*F*).

### Shedding of endogenous hPLA2R1 in podocytes is ADAM10-dependent

**Figure 9:**
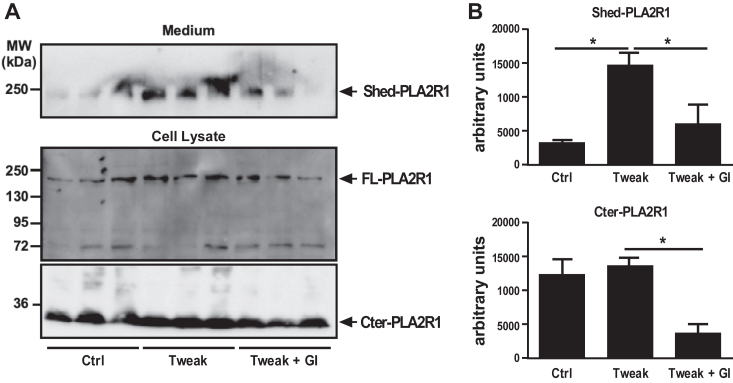
**Endogenous PLA2R1 is shed from human podocytes by an ADAM-dependent mechanism.***A*, human podocytes were pretreated for 20 min with GI254023X (10 μM) or vehicle and then with TWEAK (100 ng/ml) for 6 h. Immunoblots of medium (*upper* panel) and cell lysate (*lower* panels) were probed with anti-hPLA2R1 antibodies targeting the extracellular region of hPLA2R1 to detect Shed-PLA2R1 and FL-PLA2R1 or specific anti-Cter-PLA2R1 antibodies to detect Cter-PLA2R1. *B*, histograms showing the quantification of Shed-PLA2R1 and Cter-PLA2R1 from WB in panel *A* (n = 3, ∗*p* value < 0.05).

hPLA2R1 is endogenously expressed in cultured human podocytes, but at barely detectable levels (18, 40, 41). To demonstrate the shedding of endogenous hPLA2R1 in podocytes, we used a human podocyte cell line immortalized with the temperature-sensitive SV40-T gene (42). At the permissive temperature of 33°C, cells proliferate in a nondifferentiated state. After transfer to the nonpermissive temperature of 37°C, cells stop growing and start to express markers of *in vivo*–differentiated podocytes. In our conditions of culture, both nondifferentiated and 2-weeks differentiated podocytes expressed very little endogenous hPLA2R1. To boost the expression of hPLA2R1, we treated the podocyte cell line with the Fn14 ligand TWEAK, which has been recently shown to increase mRNA expression of hPLA2R1 in a human podocyte cell line (43). We confirmed that treatment of our differentiated podocytes for 6 h with TWEAK led to a significant increase in hPLA2R1 expression (Fig. 9). We then tested in these conditions whether shedding of hPLA2R1 does occur constitutively and can be blocked by treatment with the ADAM10 inhibitor GI254023X. Both membrane-bound (FL-PLA2R1) and soluble (Shed-PLA2R1) forms of PLA2R1 were barely detectable in podocytes untreated with TWEAK and GI254023X (Fig. 9*A* upper panel). Conversely, a short treatment with TWEAK increased FL-PLA2R1 expression in cell lysate, which was accompanied by an increase of Shed-PLA2R1 in cell medium (Fig. 9*A* middle and upper panels, respectively). In these conditions, treatment with GI254023X significantly reduced the amount of Shed-PLA2R1 (Fig. 9*B*). In cell lysate, a predominant Cter-PLA2R1 fragment was detected, the level of which was reduced by treatment with GI254023X (Fig. 9*B*). Altogether, these results show that human podocytes express endogenous hPLA2R1 in a TWEAK-inducible manner, that is then constitutively shed by proteolysis and converted into a soluble form by a GI254023X-sensitive MP that can likely be accounted for by ADAM10, as observed in HEK293 cells.

### Shedding of hPLA2R1 is increased by inflammatory stimuli

**Figure 10:**
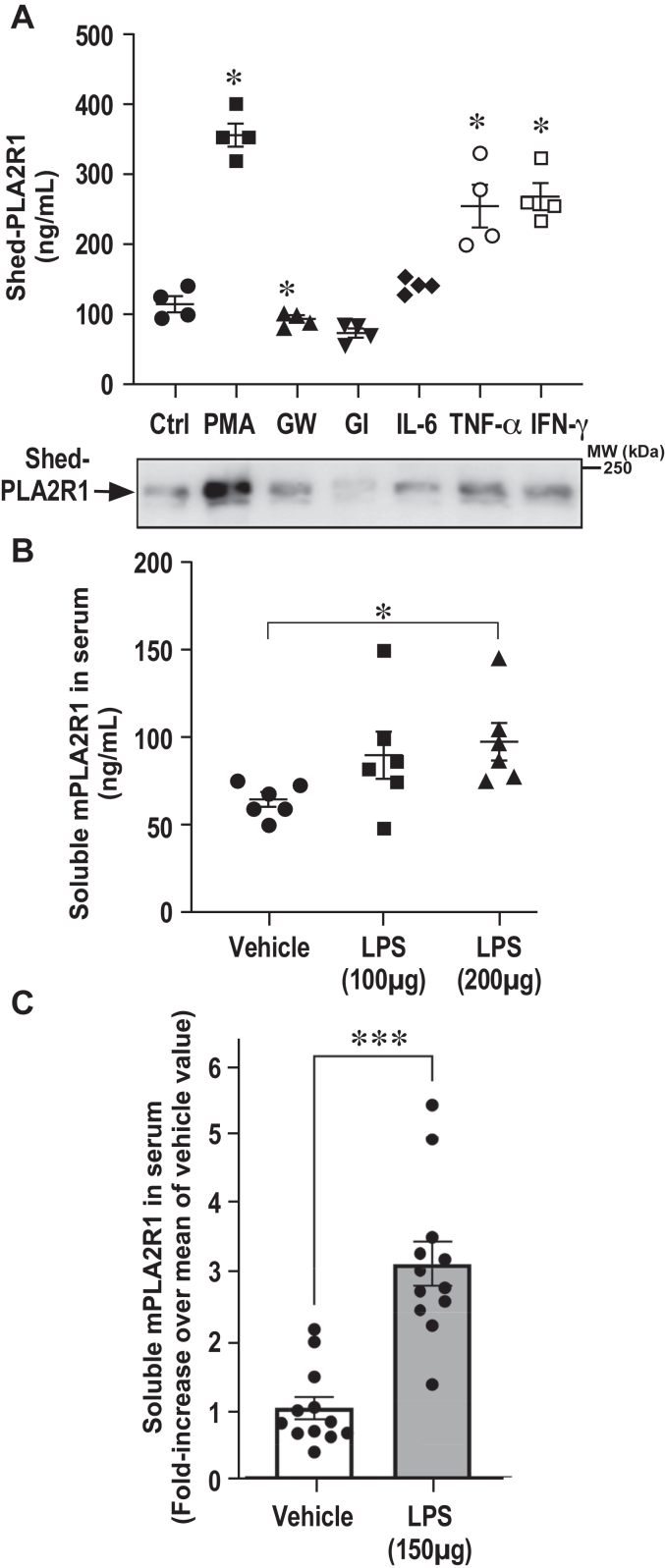
**Release of soluble PLA2R1 is triggered by inflammatory stimuli, *in vitro* and *in vivo*.***A*, hPLA2R1 expression was induced in T-Rex-293 cells by tetracycline treatment (1 μg/ml) for 48 h. Medium was changed and cells were treated for 2 h with the proinflammatory human cytokines IL-6, TNF-α, and IFNγ (20 ng/ml). The effects were compared to those of PMA (200 nM), GW280264X (GW, 10 μM), and GI254023X (GI, 10 μM). Shed-PLA2R1 present in medium was measured by TRFIA (*upper* panel, n = 4, ∗*p* value <0.05) and WB (*lower* panel) with anti-PLA2R1 antibodies targeting the extracellular region of hPLA2R1. *B*, naive adult C57BL6/J mice were injected with lipopolysaccharides (LPS, 100 or 200 μg) or vehicle. Ninety minutes later, sera were collected and the soluble form of mPLA2R1 was quantified by TRFIA (n = 6, ∗*p* value < 0.05). *C*, same experiment as in panel *B* except that 150 μg of LPS or vehicle were injected to two groups of 12 mice. Data are expressed as fold-increase of the mean value of soluble mPLA2R1 measured in serum in the absence of LPS (n = 12, ∗∗∗*p* value < 0.005).

The role of hPLA2R1 shedding by ADAM10 and ADAM17 and of soluble PLA2R1 in the pathophysiological functions of PLA2R1 are essentially unknown. It has been shown that soluble PLA2R1 inhibits sPLA2s and may act as a negative regulator of their proinflammatory role by blocking enzymatic activity (6, 8, 9, 21), while both soluble PLA2R1 and multiple sPLA2s can be induced in inflammatory conditions (3, 20, 44, 45). More generally, ADAM proteases play key roles in ectodomain shedding in autoimmune and inflammatory diseases, infections, and cancers, including kidney and podocyte diseases (28, 29, 31, 46). To provide the proof of principle of hPLA2R1 shedding along with the production of soluble PLA2R1 in inflammatory conditions, T-REx-293 cells expressing hPLA2R1 were treated for 2 h with the pro-inflammatory cytokines IL-6, TNFα, and interferon-γ (IFNγ) and their effects were compared with that of PMA and ADAM inhibitors (GI254023X, GW280264X). The level of Shed-PLA2R1 was measured in cell medium by TRFIA and WB. TNFα and IFNγ significantly increased Shed-PLA2R1 in cell medium, to levels close to that of PMA (Fig. 10*A*). Additional experiments by WB showed that stimulation by IL6, TNFα, and IFNγ was dose- and time-dependent (Fig. S6).

Finally, to expand our results in an *in vivo* context, we measured the level of circulating soluble mPLA2R1 in serum and tested the effect of injection of LPS, mimicking septic shock. C57BL/6 mice were injected with LPS or saline, and sera were collected 90 min after injection. The circulating soluble form of mPLA2R1 was quantified using a specific mPLA2R1 TRFIA assay, which was developed in-house (See Experimental procedures). The assay is sensitive and can detect recombinant soluble mPLA2R1 in the low ng/ml range (Fig. S7). The assay is specific as no signal over background was detected in the serum of mPLA2R1-deficient (*Pla2r1^−/−^*) mice (Fig. S7). A pilot experiment with six mice per group indicated that endogenous soluble mPLA2R1 is constitutively present in the serum of healthy WT mice at about 50 ng/ml and that this level is increased by injection of 100 and 200 μg of LPS (Fig. 10*B*). A second experiment with 12 mice per group, injected or not with 150 μg of LPS, confirmed that LPS induces a 3-fold increase in the level of circulating soluble mPLA2R1 (Fig. 10*C*).

Altogether, our results provide the proof-of-concept that PLA2R1 ectodomain shedding and the resulting soluble form of PLA2R1 can be modulated by inflammation induced by cytokines or LPS.

## Discussion

A soluble form of PLA2R1 has been described in the literature a few times. In 1995, our laboratory identified an mRNA splice variant coding for a soluble form of human PLA2R1, but we did not provide evidence for the presence of a soluble form in serum (5). In 2000, a soluble form of PLA2R1 was described in mouse serum, likely resulting from shedding of membrane-bound PLA2R1 by unknown MPs (20, 21). More recently, a soluble form of human PLA2R1 was found in the serum from healthy subjects (15). However, the mechanism of production of mouse and human soluble PLA2R1 was never studied.

In this study, we show that the membrane-bound form of hPLA2R1 is spontaneously cleaved within the juxtamembrane stalk, releasing a soluble secreted form called Shed-PLA2R1 and a small membrane-bound fragment of approximately 10 kDa called Cter-PLA2R1. Using multiple approaches with pharmacological inhibitors, transcriptional silencing of proteases by siRNAs, and protease-deficient cell lines, we identified PLA2R1 as a novel substrate of both ADAM10 and ADAM17, mediating ectodomain shedding of PLA2R1 and releasing its entire extracellular domain in the cell medium. Our current data obtained in three different cell types, HEK293, MEFs, and podocytes, highlight a central role of ADAM10 in basal (constitutive shedding) but also activated conditions (stimulated shedding), when cells are treated with agonists stimulating a Ca^2+^ influx, as mimicked by ionomycin in our settings, or stimulating the PKC-dependent pathway, as mimicked by PMA. ADAM17 appears to also play a role, especially in stimulated conditions, and the two proteases may act in a redundant manner in certain conditions. For example, our results by siRNA silencing indicate that constitutive shedding is mostly mediated by ADAM10, whereas PMA-induced shedding is mainly mediated by ADAM17 and only partially by ADAM10. The results obtained in HEK293 cells deficient for ADAM10, ADAM17, or both proteases provide evidence for a central role of ADAM10 in both basal and stimulated conditions. However, in these KO cell models, ADAM17 also contributes, suggesting redundant and/or compensatory mechanisms. We also provided evidence that BACE1 is not involved in the shedding of PLA2R1, at least in our experimental conditions. Furthermore, our findings cannot rule out the role of other MPs in other cells and conditions, as observed for many other transmembrane proteins undergoing shedding (47, 48). In fact, data in Figs. 6*B* and 7*D* provide preliminary evidence for shedding in HEK293 cells by at least a third protease, independent of ADAM10 and ADAM17. However, the rate of shedding by this protease appears to be much lower than that promoted by ADAM10 and ADAM17 and could only be observed in HEK293 cells deficient for both ADAMs.

Another interesting aspect of our study is that PLA2R1 does not appear to be a substrate for RIP, a highly conserved signaling pathway whereby membrane-bound proteins, in addition to “classical shedding,” are subsequently cleaved in their transmembrane region to release into the cytoplasm their cytosolic domain, which can shuttle to the nucleus to promote transcription (24, 33, 34). Our analysis using pharmacological inhibitors and cellular models deficient for presenilins/γ-secretase does not support the existence of a second cleavage of Cter-PLA2R1 by the γ-secretase protein complex. However, our findings cannot rule out the existence of RIP for PLA2R1 in other cells and/or certain physiological or pathophysiological conditions. We did not study the mechanism by which Cter-PLA2R1 disappears from cell lysate at late time points after induction of hPLA2R1 with tetracyclin (Fig. 1*C*). The potential biological role of Cter-PLA2R1, if any, remains unknown.

We studied in depth the molecular mechanism of cleavage of PLA2R1 under basal conditions, which is mostly mediated by ADAM10. Using a series of deletion mutants of the extracellular domains, we show that the large extracellular region of hPLA2R1 is dispensable for cleavage, since mutants were cleaved and produced a C-terminal fragment with an identical apparent molecular weight. An exception was the ΔF mutant, lacking both the CysR and fibronectin-like type II domain domains, which appeared to be less cleaved, as compared to other mutants. Conversely, the complete deletion of the hPLA2R1 juxtamembrane stalk between CTLD8 and the transmembrane domain abolished shedding. This stalk is conserved among orthologs of PLA2R1, and its length of 15 amino acids remains constant within the different mammalian species, suggesting that the length of the stalk between the plasma membrane and the first membrane-proximal globular domain of PLA2R1 (CTLD8) is a fundamental element of shedding (Fig. 3*C*). The length of the stalk is considered to be a major determinant of shedding, while the amino acid composition may be less important, yet this latter depends on the shed protein and the protease (24, 49, 50, 51, 52). In agreement with the importance of length, we found that deletion of six amino acids at the identified cleavage site (Δ7-Δ1388-1393) abolished the shedding of Δ7, while shorter deletions or point mutations had lower effects (Fig. S1). In further agreement, we observed that rabbit and mouse PLA2R1 were efficiently cleaved, while among PLA2R1 paralogs, hMRC1 and hMRC2 were shed at higher rate than hLY75 and have longer juxtamembrane stalks (Fig. 3*A*). To our knowledge, the detailed molecular mechanism of shedding for MRC1, MRC2, and LY75 has not been described, including the identification of the proteases involved and their respective cleavage sites.

The purification of Cter-PLA2R1 fragments from both hPLA2R1 and Δ7 followed by N-terminal sequencing unambiguously identified the cleavage site after proline 1390. This site of cleavage is likely the one preferentially targeted by ADAM10, since the experiment was performed in basal conditions where ADAM10 is centrally involved in the shedding of hPLA2R1. ADAM17 may also cleave at this position since it has been shown to cleave MerTK after proline (53), but data shown in Fig 6*B* suggest the possibility of alternative cleavage sites depending on proteases and cell activation. Despite a fairly high conservation of this residue among orthologs and paralogs of PLA2R1 (Fig. 3*C*), point mutations of proline 1390 to aspartic acid, glycine, or alanine have no or slight effect on shedding, arguing for no strict amino acid specificity at this position. These observations are in line with the literature reporting poor consensus cleavage site among shed membrane proteins (24, 52, 54). The two major sheddases, ADAM10 and ADAM17, only have broad preference for hydrophobic or aliphatic amino acids and require no specific amino acids at positions surrounding the cleavage site but rather recognize an alpha-helical structure without negatively charged residues and glycosylation within the stalk domain (52, 55, 56). Finally, it is noteworthy that the WB analysis of Cter-PLA2R1 fragments, especially those produced from the Δ7 mutant which is highly expressed in HEK293 cells (Fig. 6*B*), indicate the presence of several forms of Cter-PLA2R1 between 8 and 14 kDa. These forms appeared to be markedly increased in ADAM10 or ADAM10/ADAM17-deficient cells, suggesting the existence of alternative cleavage sites by different proteases, including shedding by MT1-MMP, meprins, or SPPL2a (57, 58).

Shedding by ADAM10 and ADAM17 is a highly regulated mechanism in multiple conditions including inflammation, cancer, and autoimmunity (24, 29, 31). In this study, we provide evidence that the shedding of PLA2R1 leading to the production of its soluble form is regulated by inflammation in different cells as well as *in vivo*. First, we show that, in HEK293 cells, the shedding of hPLA2R1 is enhanced by pro-inflammatory cytokines such as TNFα, IFNγ, and IL-6. Second, we show that, *in vivo*, injection of LPS to mice (mimicking septic shock) increases the level of circulating soluble mPLA2R1. Interestingly, ADAM17 is closely associated to inflammation, although the relationship appears very complex and dependent on the context (24, 29, 31). For example, LPS is known to rapidly activate ADAM17 through the p38 mitogen-activated protein kinase pathway (59). Moreover, ADAM17 has been implicated in the cleavage of TNFα and IL-6 receptor (60). Therefore*, in vivo*, it is conceivable that in inflammatory conditions such as endotoxic shock or in a particular inflamed microenvironment, shedding of PLA2R1 is the consequence of activation of ADAM17 which in turn cleaves PLA2R1. Third, we show that shedding of endogenous PLA2R1 occurs in podocytes stimulated with Tumor necrosis factor-like weak inducer of apoptosis (TWEAK). We confirmed the finding of Cuarental *et al.* that TWEAK upregulates the expression of endogenous PLA2R1 in cultured human podocytes (43). This in turn led to the release of the soluble form that can be blocked by GI254023X, the specific ADAM10 inhibitor, in otherwise nonstimulated conditions. Of note, TWEAK and its receptor TNFRSF12a (TNF receptor superfamily member 12a, also known as Fn14 or TWEAK receptor) are associated with various pathophysiological conditions including inflammation and cancer, but also MN (43, 61, 62). Exploration of single-cell transcriptomics and glomerular transcriptomics data identified TNFRSF12a as the most highly expressed TNF receptor superfamily gene in MN, where increased expression of PLA2R1 has also been reported (43).

Finally, our study raises the question of the physiological and pathophysiological roles of the soluble form of PLA2R1. As for many other soluble forms of membrane receptors, we can hypothesize that the soluble form of PLA2R1 acts either as a decoy receptor or promote trans-signaling, as shown for the IL-6 receptor (63). In support to a function as a decoy receptor, it was proposed that the circulating form of PLA2R1 in mouse and human serum would regulate the action of sPLA2s by inhibiting their enzymatic activity during inflammatory processes and decreasing their binding to the membrane receptor (5, 6, 8, 9, 11, 21). Several sPLA2s are overexpressed in inflammatory conditions such as in the LPS model of septic shock, suggesting an interplay between soluble PLA2R1 and sPLA2s (3, 9, 20, 44). It was also reported that recombinant soluble hPLA2R1 binds to collagen I and inhibits its interaction with the extracellular domain of integrin β1 on the surface of HEK293 cells, thereby suppressing integrin β1–mediated migratory responses to collagen I (15). By similarity with MRC1 and MRC2 paralogs, for which a soluble form has been found in serum in various conditions and plays multiple roles *via* their binding properties (64, 65), we can speculate that the soluble form of PLA2R1 may bind various known and unknown ligands to exert novel functions. PLA2R1 has also been shown to induce cellular senescence, yet the underlying molecular mechanism is unclear and may involve its soluble form (16, 17).

Last but not least, PLA2R1 has been identified as the major autoantigen in idiopathic MN (18, 19). However, nothing is known about the etiology of the disease. This includes the respective role of soluble *versus* membrane-bound PLA2R1 in the initiation and progression of the autoimmune response leading to the production of pathogenic anti-PLA2R1 antibodies (66, 67). We observed that circulating levels of the soluble form of PLA2R1 are increased in mice under inflammatory conditions induced by LPS administration. We and others hypothesize that an infectious or inflammatory event may increase the expression of PLA2R1 in tissues and cells including lung, kidney, and podocytes (although not exclusively) and in turn may lead to the release of the soluble form of PLA2R1 that plays a role in the development of the autoimmune response (19, 68, 69, 70). During the pathogenic phase, the disease is caused by the formation of immune complexes in the subepithelial space between podocytes and the glomerular basement membrane. It is commonly accepted that the immune complexes are formed by circulating antibodies that bind *in situ* to antigens intrinsically expressed at the podocyte plasma membrane, the major one being PLA2R1 (71). Interestingly, THSD7A, another autoantigen responsible for MN, as well as NEP, involved in alloimmune MN, and megalin, the podocyte autoantigen involved in the rat Heymann's nephritis MN model, share several structural similarities with PLA2R1 (72, 73, 74). In particular, they all exist as membrane-bound proteins and in a soluble form generated by shedding (75, 76, 77, 78). The Heymann's nephritis MN model suggests that binding of autoantibodies to megalin, present at the surface of podocytes, leads to shedding of the membrane-bound protein that then accumulates into immune complexes (75, 79). The antibody–megalin complex is not subject to clearance by endocytosis, instead it will cross-react with circulating antibodies and/or other immune complexes already present at the subepithelial space, gradually enlarging the immune deposits through repeated cycles of this mechanism. We can therefore hypothesize that the same occurs in PLA2R1- and THSD7A-associated MN. Of note, we show here for the first time that PLA2R1 shedding occurs endogenously in human podocytes stimulated by TWEAK. In this scenario, PLA2R1 endogenously expressed at the foot processes of the podocyte would be targeted by circulating autoantibodies leading to the formation and deposition of immune complexes *in situ*. It is noteworthy that the exact nature of the PLA2R1 form present in the immune complexes is unknown. Whether it is the soluble form of PLA2R1 released from the surface of podocytes that is trapped by autoantibodies in the glomerular basement membrane and is responsible for the immune deposits remains to be determined. ADAM10 and ADAM17 have been shown to play a key role in MN and in animal models, including increased levels of the proteases in MN biopsies, but it remains to be demonstrated which of these proteases participate to the shedding of PLA2R1 in MN (25, 29, 46).

In conclusion, our data show for the first time that PLA2R1 is a new substrate for ADAM10 and ADAM17 in several cell types and may be also a substrate for additional proteases that remain to be identified. Most of these data have been obtained *in vitro*, and they should be confirmed in an *in vivo* setting, in physiological and pathophysiological conditions. Among multiple examples of membrane proteins shed by ADAM10 and ADAM17, the overall mechanism of shedding of PLA2R1 seems to be most similar to that of the IL6 receptor, which is a very good substrate for both ADAM10 and ADAM17 (63, 80). For both proteins, shedding mediated by ADAM10 and ADAM17 has been observed, along with the presence of splice variants coding for an alternative soluble form, and finally, the two membrane proteins are also targeted to exosomes (40, 81, 82). These mechanisms likely counter-balance their rate of shedding in different biological settings.

## Experimental procedures

### Reagents

Tetracycline was from Boehringer Ingelheim. Zeocin (0.1 mg/ml) and blasticidin (5 μg/ml) were from InvivoGen. PMA and ionomycin were from Calbiochem (Merck Millipore). The protease inhibitors GM6001, TAPI-1, and DAPT and recombinant human soluble TWEAK were from Merck Millipore. GI254023X and GW280264X were from Aobious. Bi (ELN582302) and D6 (ELND006) were from Elan Pharmaceuticals. ELN582302 was synthesized based on LY2811376 (83). ELND006 was synthesized as described (84). LPS from *Escherichia coli* O127:B8 was from Sigma-Aldrich. Human IL-6, TNFα, and IFNγ were from PeproTech.

### Primary and secondary antibodies

Detection of human Shed-PLA2R1 was performed by WB under nonreducing conditions with anti-PLA2R1 antibodies from a patient with PLA2R1-associated MN (85) or anti-hPLA2R1 rabbit polyclonal antibodies. Detection of mouse (mPLA2R1) and rabbit PLA2R1 (rbPLA2R1) was performed by WB under nonreducing conditions with specific anti-mPLA2R1 rabbit polyclonal antibodies and specific anti-rbPLA2R1 guinea pig polyclonal antibodies, respectively. Anti-hPLA2R1 and anti-mPLA2R1 rabbit polyclonal antibodies were raised by immunization with the respective full extracellular region of PLA2R1 produced in-house (9, 86). Anti-rbPLA2R1 guinea pig polyclonal antibodies were raised by immunization with the full extracellular region of rabbit PLA2R1 (10). Specific anti-Cter-PLA2R1 rabbit polyclonal antibodies were raised by immunization with a DsbC fusion protein containing the Cter-PLA2R1 intracellular domain, which was produced in-house as described for other PLA2R1 domains (85). Immunization of rabbits was performed with standard immunization protocols by Covalab. Antibodies against hLY75 were used under nonreducing conditions to detect hLY75 (ExBio). Antibodies against hMRC2 were used under nonreducing conditions to detect hMRC2 (R&D systems). Antibodies against hMRC1 (Atlas Antibodies) were used under reducing conditions to detect hMRC1. Mouse monoclonal anti-HA, anti-Flag, and anti-ß-actin antibodies (Merck Sigma-Aldrich) were used under reducing conditions. Anti-HSP60 antibodies (Santa Cruz Biotechnology) were used under reducing conditions. Anti-ADAM10 antibodies and Anti-ADAM17 antibodies (Abcam) were used under reducing conditions. Anti-PS1 N-terminal fragment antibodies and anti-PS2 C-terminal antibodies were generous gifts from Dr Paul Fraser (Tanz Centre for Research in Neurodegenerative Diseases, University of Toronto) and Dr Nobuo Araki (Saitama Medical University), respectively. Goat anti-mouse secondary antibodies coupled with HRP were from Jackson ImmunoResearch. Goat anti-human IgG4 secondary antibodies coupled with HRP were from Southern Biotech. Goat anti-human IgG secondary antibodies coupled with Alexa488 and goat anti-rabbit IgG secondary antibodies coupled with Alexa488 were from Gibco (Thermo Fisher Scientific).

### Cell culture

Except otherwise indicated, cells were grown at 37 °C and 5% CO_2_ in a humidified incubator. Basal culture medium for HEK293 cells was high glucose Dulbecco’s modified Eagle’s medium, supplemented with 10% heat-inactivated fetal bovine serum, 50 units/ml penicillin G, 100 μg/ml streptomycin (all from Gibco, Thermo Fisher Scientific). The tetracycline-inducible T-REx-293 cell line was from Invitrogen (Thermo Fisher Scientific). HEK293 cells, WT or deficient for ADAM10, ADAM17, or both proteases, were generated using the CRISPR/Cas9 system (38). MEFs deficient for ADAM10, ADAM17, or the presenilin/γ-secretase complex were provided generously by Pr. Saftig (Institute for Biochemistry, Christian-Albrechts-University of Kiel), Dr R. Black (Immunex), and Pr. Bart de Strooper (University of Leuven), respectively. The immortalized human podocyte cell line LY was a generous gift from Pr. Moin Saleem (University of Bristol) (42). Cells were immortalized with the temperature-sensitive SV40 gene. Podocytes proliferate at the permissive temperature of 33 °C. For differentiation, podocytes are plated on collagen-coated dishes and transferred to the nonpermissive temperature of 37 °C for 15 days, during which they undergo growth arrest and express markers of podocyte differentiation. Podocytes were grown in RPMI-1640 supplemented with 10% heat-inactivated fetal bovine serum, 50 units/ml penicillin G, 100 μg/ml streptomycin, and insulin-transferrin-selenium supplement (all from Gibco, Thermo Fisher Scientific).

### Generation of T-Rex-293 cells stably expressing WT hPLA2R1

T-Rex-293 cells overexpressing hPLA2R1 with a tetracycline-regulated expression system were obtained as previously described (87). The cDNA coding for membrane-bound hPLA2R1 harboring a C-terminal HA tag (hPLA2R1-HA) was inserted into the pcDNA4/TO expression vector (InvitroGen) (see Table S1 for more details). The expression plasmid was transfected into T-Rex-293 cells with Lipofectamine 2000. The cells were cotransfected with a pcDNA6/TR plasmid coding for the bacterial tetracycline resistance operon, which activates the transcription of hPLA2R1 when cells are cultured in the presence of tetracycline. Cells expressing both plasmids were selected with zeocin (100 μg/ml) and blasticidin (5 μg/ml). hPLA2R1 expression was induced with tetracycline (1 μg/ml).

### Expression plasmids for hPLA2R1 mutants, orthologs, and paralogs

The series of membrane-bound hPLA2R1 deletion mutants with a C-terminal HA tag were generated as described previously (85). The Δ7 and Δ7ΔShed mutants were produced with an N-terminal 3xFlag tag to facilitate detection of the small shed fragment (Shed-Δ7 or CTLD8). Constructs for hPLA2R1 ΔShed and for Δ7 mutants with point mutations at proline 1390 or deletions within the juxtamembrane stalk region were generated by reverse PCR, using as template hPLA2R1 or Δ7 constructs. More details about all of these constructs are presented in Table S1. Rabbit and mouse PLA2R1 expression plasmids were generated as previously described (1, 9). Full-length cDNA coding for human macrophage mannose receptor (hMRC1, Uniprot P22897) and human endocytic receptor 180 (hMRC2, Uniprot Q9UBG0) were cloned by PCR using standard methods. The cDNA coding for human LY75 (DEC-205, Uniprot O60449) was purchased from GeneCopoeia. All cDNA constructs were verified by sequencing after subcloning into the mammalian expression vectors.

### Transient transfection of hPLA2R1, mutants, orthologs, and paralogs in HEK293 and MEFs

Transient transfection of all above expression plasmids for hPLA2R1 WT, deletion and point mutants, orthologs and paralogs were performed in WT HEK293 cells using the calcium/phosphate method or Lipofectamine 2000 (Invitrogen, Thermo Fisher Scientific) according to the manufacturer’s instructions. Expression of full-length forms of orthologs and paralogs were validated by WB using anti-HA antibodies and their specific antibodies (see above). For HEK293 cells deficient for ADAM10, ADAM17, or both, cells were seeded in 12-well plates (100,000 cells/well) and transfected with expression plasmids encoding for hPLA2R1 with a C-terminal HA tag or the Δ7 mutant with a N-terminal 3xFlag tag and a C-terminal HA tag. Cells were used for shedding experiments 48 h post-transfection. For MEFs deficient for ADAM10, ADAM17, or presenilins, cells were seeded in 6-well plates (200,000 cells/well) and transfected with the Δ7 mutant using Lipofectamine 2000. Cells were used for shedding experiments 24 h after transfection. The expression of recombinant proteins was validated by WB using anti-HA, anti-Flag, or protein-specific antibodies.

### Immunocytofluorescence of hPLA2R1 expressed in T-REx-293 cells

T-REx-293 cells stably expressing hPLA2R1-HA were seeded in 12-well plates (20,000 cells/well) on poly-D-lysine–coated glass coverslips (Sigma-Aldrich). Twenty-four hours later, cells were treated with vehicle or tetracycline (1 μg/ml) for 48 h. Cells were fixed for 20 min with 4% paraformaldehyde, rinsed with PBS^+ +^ (containing Mg^2+^ and Ca^2+^, Gibco, Thermo Fisher Scientific), and incubated at 4 °C for 1 h in PBS^++^ containing 0.5% bovine serum albumin (BSA). Cells were then incubated at 4 °C for 1 h with anti-PLA2R1 antibodies from a patient with PLA2R1-associated MN (85) (1/200, in PBS^++^ with 0.5% BSA). After three washes with PBS^++^, cells were incubated with goat anti-human IgG secondary antibodies coupled with Alexa Fluor 488 dye (A488, 1/500, in PBS^+^ containing 0.5% BSA and 0.5% goat serum) for 1 h at room temperature. After washing, DAPI (Invitrogen, Thermo Fisher Scientific) was added to cells and incubated for 10 min. Coverslips were mounted on glass slides with Fluoroshield mounting medium (Sigma-Aldrich), air dried, and imaged with a confocal microscope equipped with the FV10-ASW viewer software (Olympus, https://www.olympus-lifescience.com/en/downloads/detail-iframe/?0[downloads][id]=847249651).

### Staining of hPLA2R1 in T-REx-293 cells by flow cytometry

T-REx-293 cells stably expressing hPLA2R1-HA were induced with tetracycline (1 μg/ml) for 48 h. Control cells received vehicle. Cells were collected in PBS containing 1 mM EDTA and 0.5% BSA and fixed for 20 min with 4% paraformaldehyde. After centrifugation, cells were resuspended in a blocking solution (PBS containing 0.5% BSA, 1 mM EDTA, 5% FBS) for 30 min at room temperature. After centrifugation, cells were labeled with anti-hPLA2R1 rabbit polyclonal antibodies or pre-immune serum (1/500, in PBS containing 0.5% BSA, 1 mM EDTA) for 45 min at room temperature. After three washes, cells were labeled with goat anti-rabbit secondary antibodies coupled with Alexa488 (1/500 in PBS containing 0.5% BSA, 1 mM EDTA) for 45 min. Cells were washed three times and analyzed by flow cytometry with a LSR Fortessa instrument (BD Biosciences).

### Transfection of siRNA and validation of gene interference for ADAM10 and ADAM17 by RT-qPCR

siRNA directed against ADAM10 or ADAM17 or scrambled siRNA (QIAGEN) were transfected in hPLA2R1-expressing T-REx-293 cells using RNAiMax Lipofectamine (Invitrogen, Thermo Fisher Scientific) according to the manufacturer’s instructions. To maximize the decrease of ADAM expression at the time of hPLA2R1 expression and shedding, cells were transfected with siRNAs at day 0, induced with tetracyclin at day 1, and tested for shedding at day 2, in basal or stimulated conditions. To validate gene interference, total RNA from siRNA-transfected HEK293 cells were isolated using the Trizol RNA extraction kit (Invitrogen, Thermo Fisher Scientific) according to the manufacturer’s instructions, followed by treatment of total RNA with RQ1 DNAse (Promega). First-strand cDNA was synthesized from 2 μg of total RNA with 200 U of SuperScript III reverse transcriptase (Invitrogen, Thermo Fisher Scientific) in the presence of 25 μg/ml random primers, 0.5 mM deoxyribonucleotide triphosphate mix, 5 mM DTT, 40 U RNAsin (Promega). The reaction was incubated for 5 min at 25 °C, then 50 min at 50 °C, then inactivated for 15 min at 70 °C. Quantitative PCR was performed using the SYBRgreen method (Roche Diagnostics) with the LightCycler 480 real-time PCR system (Roche Diagnostics). β-actin and GAPDH were used as reference genes for normalization. Primers were from QIAGEN (QuantiTect primer assay, QIAGEN).

### Shedding assays

In most assays, cells were seeded in 12- or 6-well plates. hPLA2R1-expressing T-REx-293 cells were induced with tetracycline for 48 h. In other cases, cells were transiently transfected with the different expression plasmids as described above. Forty-eight hours after transfection, medium was changed for serum-free Opti-MEM I (Gibco, Thermo Fisher Scientific), and cells were left untreated, stimulated with PMA (200 nM) or ionomycin (2.5 μM) for various times, and/or pretreated for 30 min with the different inhibitors (Gi 10 μM, DAPT 10 μM, Bi 100 μM, D6 12.5 μg/ml, or other concentrations as indicated in figure legends) before stimulation at 37 °C. Cell media were collected and centrifuged for 30 min at 20,000*g* (except in Fig. S1*A* where cell medium was centrifuged at various centrifugal forces) before analysis by WB or TRFIA. Cells were rinsed and detached in PBS before centrifugation. To prepare cell lysate, cell pellets were lysed in RIPA buffer (50 mM Tris–HCl pH 8.0, 150 mM NaCl, 0.1% SDS, 1% Triton X-100, 0.5% sodium deoxycholate) containing 1X protease inhibitor cocktail (Roche Diagnostics), sonicated for 15 s, incubated at 4 °C for 1 h on a rotative wheel, and centrifuged for 30 min at 20,000*g*. The clarified supernatant was used as cell lysate for WB analysis.

### WB analysis

Protein samples from cell medium containing the soluble forms of hPLA2R1, deletion mutants, orthologs and paralogs, and protein samples from cell lysate containing the membrane-bound forms (cleaved or not) were analyzed by SDS-PAGE under nonreducing or reducing conditions depending on the specificity of antibodies. For some orthologs and paralogs of hPLA2R1, various volumes of cell medium were precipitated with 10% trichloroacetic acid for 1 h at 4 °C before loading. Proteins were separated by SDS-PAGE using the most appropriate percentage of acrylamide, for instance 7% polyacrylamide gel for FL-hPLA2R1 and Shed-PLA2R1, discontinuous 6 to 12% to visualize both FL-PLA2R1 and Cter-PLA2R1, 4 to 20% gradient gel for large deletion mutants and for orthologs and paralogs, and 14 to 16% Tris-Tricine gel for Δ7 mutants and specific analysis of Cter-PLA2R1 fragments. For immunoblotting, proteins were transferred to methanol-soaked polyvinylidene difluoride blotting membranes (Bio-Rad) under semi-dry conditions using a trans-blot turbo transfer system (Bio-Rad) at 25 V constant voltage for 30 min. Alternatively, 16% Tris-Tricine gels were directly transferred to nitrocellulose blotting membranes (Amersham Protran 0.45 μm NC, GE Healthcare Life Science) using a mini trans-blot cell transfer system (Biorad) at 65 V constant voltage for 2 h. Membranes were blocked in 5% milk in PBS for 1 h at room temperature, incubated overnight with primary antibodies at 4 °C, and then incubated for 1 h at room temperature with secondary antibodies after washing steps. Immunoreactive proteins were visualized using an enhanced chemiluminescent system (Western Lightning ECL Pro, Revvity) following the manufacturer's instructions and imaged with the Fusion FX Vilber imaging system. Western blot bands were quantified by gray value analysis using ImageJ software (https://imagej.net/ij/). WB shown in figures are one representative experiment among at least two experiments with similar results. WB with antibodies against β-actin or HSP60 served as loading controls.

### Identification of the hPLA2R1 cleavage site by Edman sequencing

Large cultures of HEK293 cells (about 10^8^ cells from multiple 15-cm Petri dishes) were transiently transfected with expression plasmids coding for hPLA2R1 or the Δ7 mutant with a C-terminal HA tag using the calcium phosphate method. Cells were not stimulated with PMA or ionomycin. Forty-eight hours later, cells were washed with PBS, scraped in lysis buffer (20 mM Tris–HCl pH 7.4, 2 mM EDTA, and 1X protease inhibitors (Roche)), lysed by sonication, and centrifuged for 30 min at 100,000*g*. Pellets containing crude cell membranes were resuspended at 2.5 mg/ml of total protein in solubilization buffer (50 mM Tris–HCl pH 7.4, 100 mM NaCl, 2 mM EDTA, 0.5% Triton X-100, 0.5% sodium deoxycholate, 1X protease inhibitors), sonicated for 10 s, and then incubated at 4 °C for 1 h to allow for maximal solubilization of membrane proteins. The solutions were centrifuged for 45 min at 100,000*g*. The supernatants containing solubilized proteins were incubated overnight at 4 °C with monoclonal anti-HA agarose beads (Sigma-Aldrich). Full-length proteins and their Cter-PLA2R1 fragments were eluted in a solution of 0.1 M glycine, pH 2.5 containing 0.3% n-octyl glucoside. Analytical amounts of purified proteins were separated on 10 to 20% Tris-Tricine SDS-PAGE precast gels (Bio-Rad) and analyzed by silver staining, Coomassie brilliant blue staining, and WB with anti-HA antibodies to visualize the full-length forms of hPLA2R1 and Δ7, as well as their Cter-PLA2R1 fragments. Preparative amounts of purified proteins were similarly separated on Tris-Tricine gels pre-run with 0.2 mM sodium thioglycolate added to the cathode buffer to minimize blocking of the N-terminal amino acid sequence, blotted on high capacity Problott PVDF membranes (Applied Biosystems), and stained with Coomassie brilliant blue according to the manufacturer’s instructions. The Cter-PLA2R1 fragments were excised from the Problott membrane and subjected to Edman sequencing. PVDF strips containing the Cter-PLA2R1 fragments were rinsed three times with Ethanol/water (90/10), dried, and loaded on the cartridge of a Procise 494A sequenator (Applied Biosystems). Protein sequencing according to the Edman’s degradation method was carried out, using the “2-cart PL PVDF-Protein” program.

### TRFIA to detect the soluble forms of hPLA2R1 and mPLA2R1

Specific TRFIAs to detect the soluble forms of hPLA2R1 and mPLA2R1 proteins were developed as described earlier for human sPLA2s (88) with modifications. Here, the TRFIA sandwich assays use specific rabbit polyclonal antibodies targeting the human PLA2R1 soluble form (anti-hPLA2R1) or the mouse soluble form (anti-mPLA2R1) obtained after three rounds of immunization of rabbits (Covalab) with the full extracellular domain of hPLA2R1 or mPLA2R1, respectively. The IgG fraction of polyclonal antibodies are used for both the coating and detection steps, assuming several epitopes are targeted in each antigen, allowing the formation of a sandwich with the antigen. The specificity of the antisera for hPLA2R1 and mPLA2R1 was verified using indirect ELISA and WB under nonreducing conditions. To purify the rabbit total IgG fraction used for coating (see below), rabbit antiserum (10 ml) was diluted twice in PBS and loaded onto a 2 × 1 ml HiTrap Protein G HP column (Merck) according to the manufacturer’s instructions. PBS was used as binding and washing buffer and glycine (0.1 M, pH 2.5) as elution buffer. The quantity, purity, and integrity of purified total IgG were verified by SDS-PAGE followed by Coomassie brilliant blue staining. To prepare anti-PLA2R1 antibodies as detection antibody, a fraction of purified rabbit IgGs was biotinylated using EZ-link NHS-Biotin (Thermo Fisher Scientific) and purified on a PD-10 column (Merck) according to the manufacturer’s instructions.

To run specific TRFIA assays, 96-well microtitration plates (Delfia plate, PerkinElmer) were coated with anti-PLA2R1 rabbit total IgG (1 μg/well) diluted in sodium phosphate (0.1 M, pH 4.9) as capture antibody and incubated overnight at 4 °C under shaking. Blocking was performed with Tris–HCl (50 mM pH 7.8) buffer containing 0.9% NaCl, 1% BSA, 6% D-sorbitol, 0.05% NaN_3_, and 1 mM CaCl_2_ for at least 2 h at room temperature under shaking. After washing with Tris–HCl (50 mM pH 7.8) buffer containing 0.9% NaCl, 0.05% NaN_3_, and 0.02% Tween20, analytes (cell medium or serum-containing soluble forms of PLA2R1 or PLA2R1 standards) were diluted in a Tris–HCl (50 mM pH 7.8) assay buffer containing 0.5% BSA, 0.05% NaN_3_, 0.01% Tween40, and 20 μM diethylenetriaminepentaacetic acid and added to wells for 1 h at room temperature under shaking. After washing, biotinylated rabbit total IgG (0.1 μg/well, diluted in assay buffer) was added and incubated for 1 h at room temperature under shaking. After washing, detection was performed by incubation with Europium-labeled streptavidin (1/1000 working dilution in assay buffer, 100 μl/well, PerkinElmer) for 1 h at room temperature under shaking. Finally, after washing, Delfia enhancement solution (PerkinElmer, Waltham) was incubated for 5 min with shaking as above and fluorescence counts were measured using a PerkinElmer EnVision Multilabel plate reader (PerkinElmer) with excitation and emission wavelengths of 340 nm and 615 nm, respectively. Standard curves were established using recombinant purified soluble forms of hPLA2R1 or mPLA2R1, and data were plotted using the GraphPad Prism software (https://www.graphpad.com/). Negative and positive controls were used to determine background and cut-off values for positivity.

### Mice

WT C57BL/6J mice were purchased from the Jackson Laboratory or Janvier Labs. mPLA2R1 KO (*Pla2r1*^−/−^) mice were obtained as described (17). All experiments were performed with 8- to 12-week-old female mice. Mice were housed on a 12 h light/dark cycle (lights on/off at 7:00/19:00) with food *ad libitum*. All animals were handled in accordance with our local Animal Care and Use Committee guidelines. All animal procedures were approved by the French Ministry of Higher Education, Research and Innovation (MESR agreement APAFIS#43724-2023030717307262).

### Detection of soluble mPLA2R1 in mouse serum from LPS-treated mice

WT female C57BL6/J mice were treated by intraperitoneal injection with saline or LPS (100, 150 or 200 μg/mouse, freshly dissolved in sterile saline prior to injection). Ninety minutes later, mice were sacrificed by cervical dislocation. Serum samples were separated from whole blood, which was collected by intracardiac puncture. Soluble mPLA2R1 was quantified by TRFIA as described above.

### Statistical analysis

Statistical analysis was performed using the GraphPad Prism software. Significant differences between two groups of data were determined using the Mann-Whitney test for nonparametric data. Alternatively, the Kruskal–Wallis test followed by a Conover-Iman test was used for multiple comparisons with Bonferroni correction between two independent groups. Results from data analysis were expressed as mean ± SEM. Statistical significance was set at ∗*p* value < 0.05 and ∗∗*p* value < 0.01, ∗∗∗*p* value < 0.005.

## Data availability

All data are contained within the manuscript.

## Supporting information

This article contains supporting information.

## Conflict of interest

The authors declare that they have no conflicts of interest with the contents of this article.
