# Supplementary material for: Ectodomain shedding of PLA2R1 is mediated by the metalloproteases ADAM10 and ADAM17
Source: J Biol Chem. 2024 Jun 17;300(7):107480. doi: 10.1016/j.jbc.2024.107480 (PMC11301074; doi:10.1016/j.jbc.2024.107480)
Supplement: Supplemental Figures S1–S7 and Table S1 [file mmc1.pdf]

SUPPORTING INFORMATION

Ectodomain shedding of PLA2R1 is mediated by the metalloproteases ADAM10 and ADAM17

Guillaume Dolla[1], Sarah Nicolas[1], Ligia Ramos dos Santos[2], Alexandre Bourgeois[2], Raphaëlle Pardossi-Piquard[2], Franck Bihl[1], Christelle Zaghrini[1], Joana Justino[1], Christine Payré[1], Pascal Mansuelle[3], Christoph Garbers[4], Pierre Ronco[5,6], Frédéric Checler[2], Gérard Lambeau[1*#], Agnès Petit-Paitel[1*#]

[1]Université Côte d'Azur (UniCa), Centre National de la Recherche Scientifique, Inserm, Institut de Pharmacologie Moléculaire et Cellulaire, Sophia Antipolis, Valbonne, France.

[2]Université Côte d'Azur (UniCa), Centre National de la Recherche Scientifique, Inserm, Institut de Pharmacologie Moléculaire et Cellulaire, Laboratoire d'Excellence DistALZ, Sophia Antipolis, Valbonne, France.

[3]Plateforme de Protéomique de l'Institut de Microbiologie de la Méditerranée (IMM), Marseille Protéomique (MaP), Aix Marseille Université (AMU), Centre National de la Recherche Scientifique (CNRS) FR3479, 31 Chemin Joseph Aiguier, 13009 Marseille, France.

[4] Institute of Clinical Biochemistry, Hannover Medical School, Hannover, Germany

[5] Institut National de la Santé et de la Recherche Médicale (INSERM), UMR-S1155, Paris, France.

[6] Sorbonne Université, Université Pierre et Marie Curie Paris 06, Paris, France.

* Contributed equally as last authors.

[#] To whom correspondence should be addressed: Gérard Lambeau (lambeau@ipmc.cnrs.fr) or Agnès Petit-Paitel (apetit@ipmc.cnrs.fr), Institut de Pharmacologie Moléculaire et Cellulaire, UMR 7275 CNRS, Université Côte d'Azur et Inserm, 660 Route des Lucioles, Sophia-Antipolis, 06560 Valbonne, France.

Running title: PLA2R1 shedding by ADAM10 and ADAM17

Keywords: PLA2R1, soluble PLA2R1, shedding, metalloproteases, ADAM10, ADAM17, membranous nephropathy, podocyte, inflammation

A

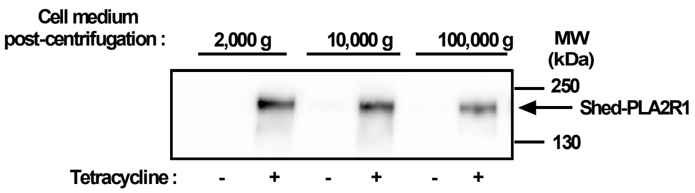

B

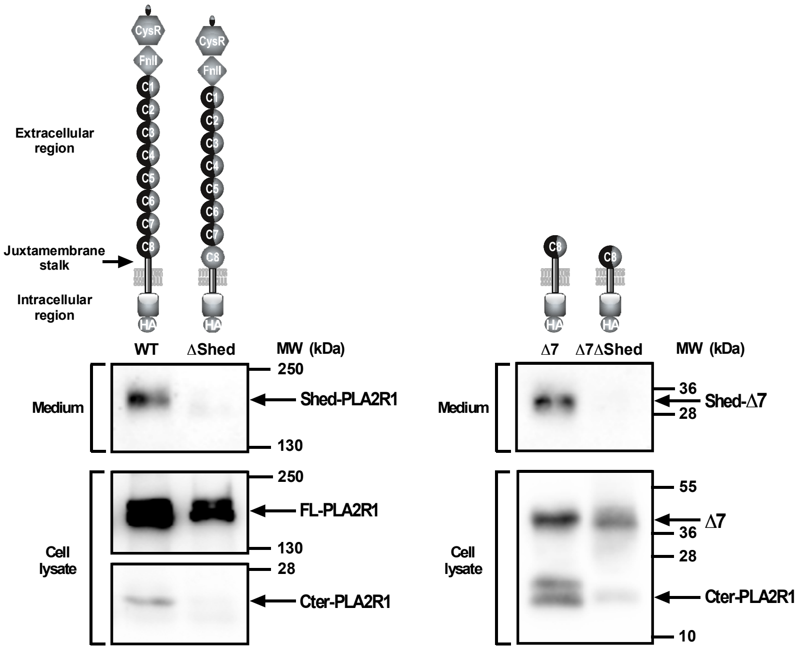

C

CTLD8 | **Juxtamembrane Stalk** | *Transmembrane Domain*

cleavage site

```
PLA2R1-Human      ...GFICKMEADIHTAEALPEKGPSHSIIPLAVVLTLIVIVAICTLSFCIYKHN...

Δ7-Δ1388-1393     ...GFICKMEADIHTAE------PSHSIIPLAVVLTLIVIVAICTLSFCIYKHN...
Δ7-Δ1391-1393     ...GFICKMEADIHTAEALP---PSHSIIPLAVVLTLIVIVAICTLSFCIYKHN...
Δ7-Δ1388-1390     ...GFICKMEADIHTAE---EKGPSHSIIPLAVVLTLIVIVAICTLSFCIYKHN...
Δ7-P1390D         ...GFICKMEADIHTAEALDEKGPSHSIIPLAVVLTLIVIVAICTLSFCIYKHN...
Δ7-P1390G         ...GFICKMEADIHTAEALGEKGPSHSIIPLAVVLTLIVIVAICTLSFCIYKHN...
Δ7-P1390A         ...GFICKMEADIHTAEALAEKGPSHSIIPLAVVLTLIVIVAICTLSFCIYKHN...
Δ7                ...GFICKMEADIHTAEALPEKGPSHSIIPLAVVLTLIVIVAICTLSFCIYKHN...
Δ7-ΔShed          ...GFICKMEAD---------------IIPLAVVLTLIVIVAICTLSFCIYKHN...
                     *********               **************************
```

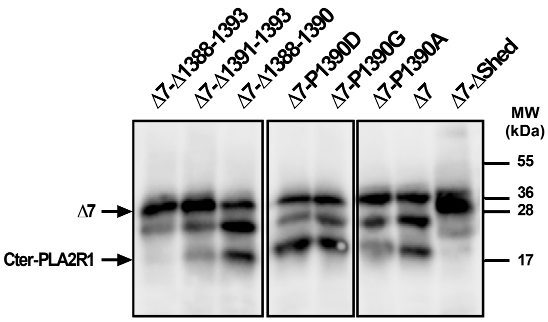

Supplementary Fig S1

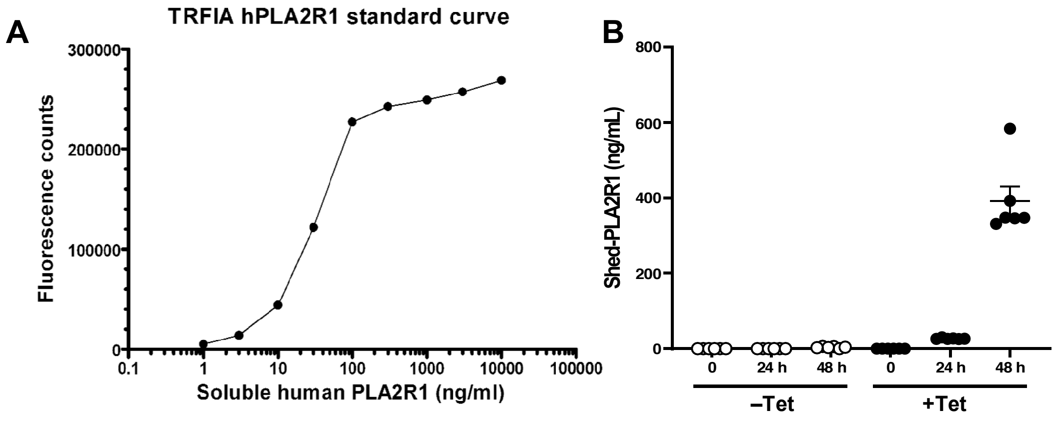

Supplementary Fig S2

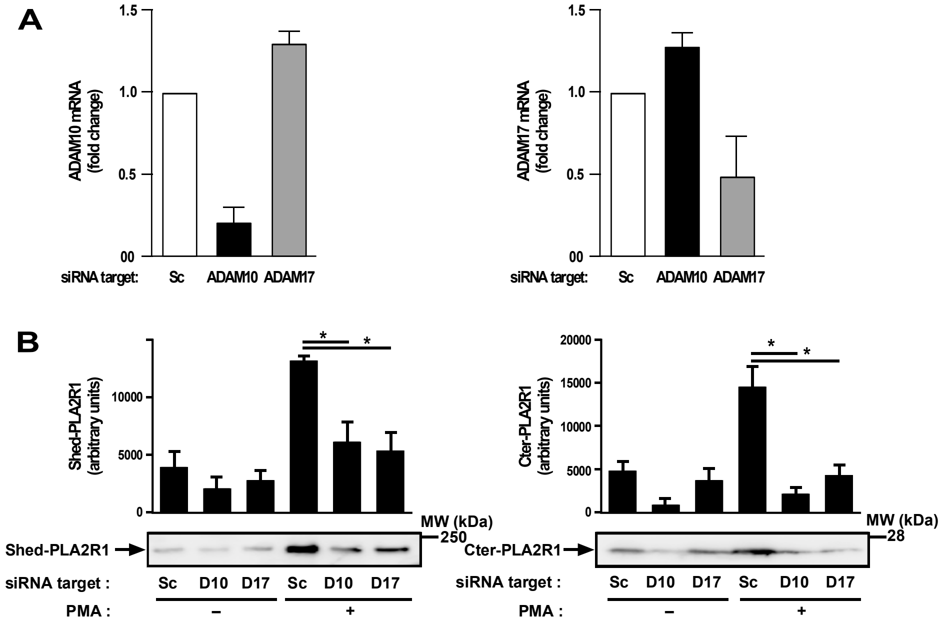

Supplementary Fig S3

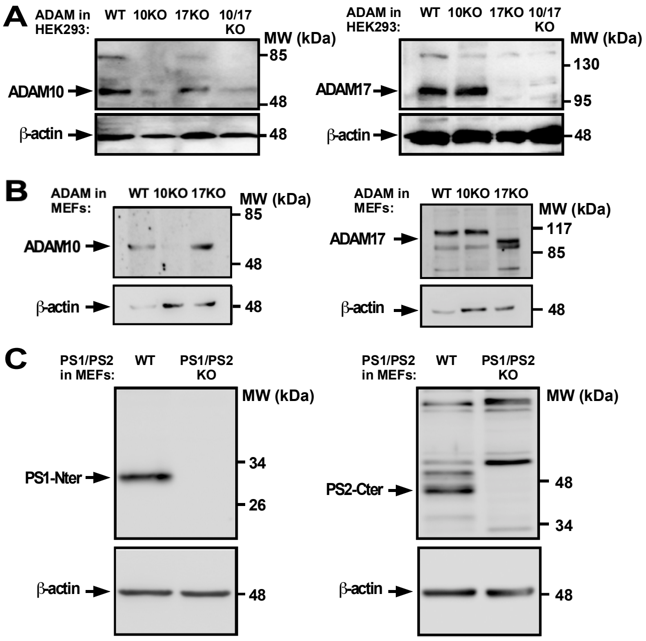

Supplementary Fig S4

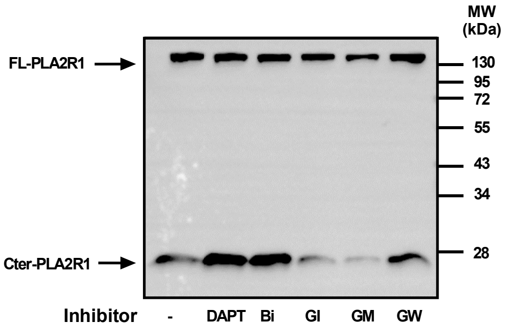

Supplementary Fig S5

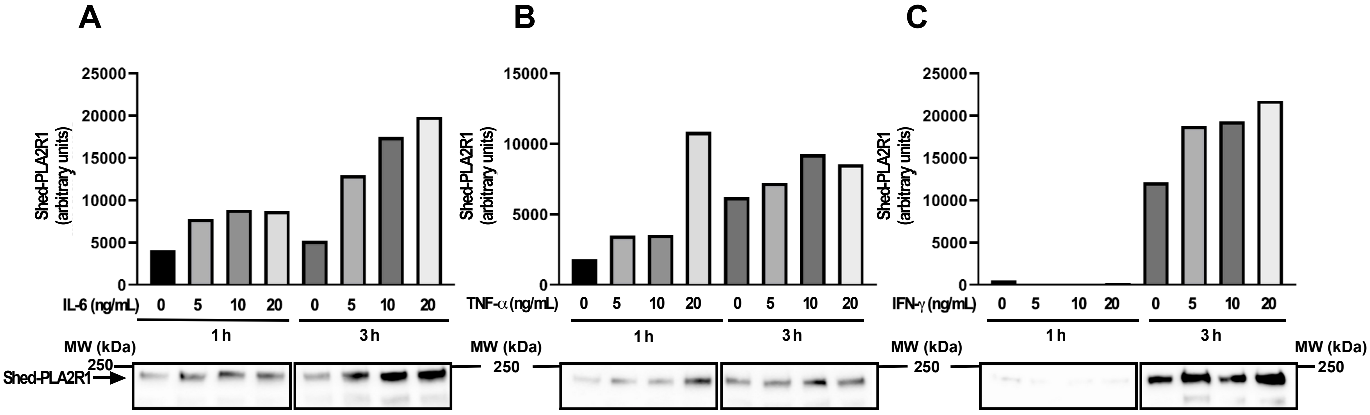

Supplementary Fig S6

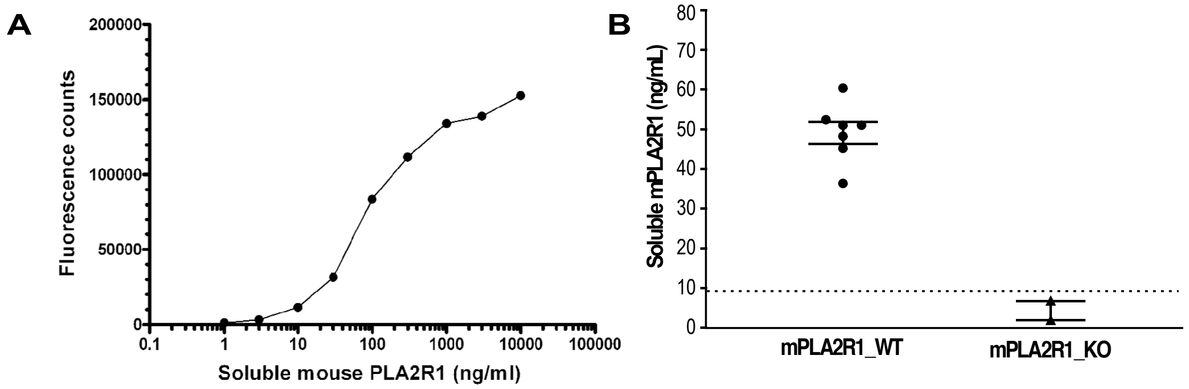

Supplementary Fig S7

Legend to Figures S1 to S7

Supplementary Figure S1: Analysis of Shed-PLA2R1 in cell medium after high-speed centrifugation and analysis of various mutants at the juxtamembrane cleavage site. ***A, Cell medium from T-REx™-293 cells stably expressing hPLA2R1 contains soluble PLA2R1 (Shed-PLA2R1), even after high-speed centrifugation.*** Cell medium from T-REx™-293 cells stably expressing hPLA2R1 treated with vehicle (left panel) or 1 µg/ml tetracycline for 48 h was centrifuged at various centrifugal forces for 60 minutes, and then analyzed by WB with anti-hPLA2R1 antibodies. The same volume of medium was loaded in each lane. ***B, hPLA2R1 ΔShed and Δ7ΔShed mutants are not cleaved in HEK293 cells. WT versus hPLA2R1 ΔShed and Δ7 versus Δ7ΔShed were transfected in HEK293 cells.*** Three days after transfection, cell lysates were prepared and equal amounts of protein samples were analyzed by WB with anti-HA antibodies. Only trace amounts of Cter-PLA2R1 were detected for the hPLA2R1 ΔShed and Δ7ΔShed mutants. The corresponding soluble forms (Shed-PLA2R1 and Shed-Δ7) were detected in cell medium using anti-hPLA2R1 specific antibodies, showing a strong reduction in the amounts of soluble forms for the hPLA2R1 ΔShed and Δ7ΔShed mutants versus WT and Δ7, respectively. ***C, Deletions and point mutations within the hPLA2R1 proteolytic cleavage site impact on shedding of the Δ7 mutant.*** Three C-terminally HA-tagged deletion mutants of Δ7 at, after or before the cleavage site (Δ1388-1393, Δ1391-1393, Δ1388-1390), three single point mutants at the proline cleavage site (P1390D, P1390G, P1390A) and Δ7 and Δ7ΔShed mutants (as controls) were designed and transfected in HEK293 cells. Three days after transfection, cell lysates were prepared and equal protein samples were analyzed by WB with anti-HA antibodies.

Supplementary Figure S2: Quantification of soluble hPLA2R1 in cell medium by Time-Resolved FluoroImmunoAssay (TRFIA). ***A***, Standard curve with recombinant purified soluble hPLA2R1 generated by plotting the fluorescence counts versus the concentration of soluble hPLA2R1 from 0 to 10,000 ng/mL (Log scale). ***B***, Concentrations of hPLA2R1 in cell medium from T-REx™-293 cells stably expressing hPLA2R1 treated with vehicle (left panel) or 1 µg/ml tetracycline for 48 h as measured by TRFIA.

Supplementary Figure S3: Gene silencing of ADAM10 and ADAM17 by siRNA suggests a role of both ADAM10 and ADAM17 in the constitutive and stimulated shedding of hPLA2R1 in HEK293 cells. ***A***, T-REx™-293 cells stably expressing hPLA2R1 were transfected with scrambled (Sc) or specific siRNAs for ADAM10 or ADAM17. Histograms show RT-qPCR analyses of the fold-change in mRNA expression for ADAM10 (left panel) and ADAM17 (right panel) 48 h post-transfection. ***B***, T-REx™-293 cells stably expressing hPLA2R1 were transfected with scrambled (Sc) or specific siRNAs for ADAM10 or ADAM17 for 48 h. hPLA2R1 expression was induced by treatment with tetracycline

(1 µg/ml) for 24 h. Medium was then changed and cells were treated with vehicle or PMA (200 nM) for one hour. Protein samples from medium (left panel) and cell lysate (right panel) were analyzed by WB (a representative experiment is shown). Blots were probed with anti-PLA2R1 antibodies targeting the extracellular region of hPLA2R1 to detect Shed-PLA2R1 (left panel) or anti-HA antibodies to detect Cter-PLA2R1 (right panel). Histograms show the quantification of 3 independent experiments (n=3, * P value <0.05).

Supplementary Figure S4: Validation of knock-down of ADAM10, ADAM17 and presenilins in HEK293 cells and mouse embryonic fibroblasts. ***A***, WB analysis of human ADAM10 (left panel) and ADAM17 (right panel) in HEK293 cells either wild-type (WT), or deficient for ADAM10 (10KO), ADAM17 (17KO) or both proteases (1017KO). ***B***, WB analysis of mouse ADAM10 (left panel) and ADAM17 (right panel) in mouse embryonic fibroblasts (MEFs) either wild-type (WT), or deficient for ADAM10 (10KO) or ADAM17 (17KO). ***C***, WB analysis of presenilin 1 (PS1, left panel) and presenilin 2 (PS2, right panel) expression in mouse embryonic fibroblasts (MEFs) either wild-type (WT) or deficient for presenilins (PS1/PS2 KO). Actin was used as loading control in all panels.

Supplementary Figure S5: Treatment with DAPT and Bi for 6 h does not affect the shedding of hPLA2R1 in HEK293 cells. hPLA2R1 expression was induced in T-REx™-293 cells by tetracycline treatment (1 µg/ml) for 48 h. Medium was changed and cells were treated for 6 h with vehicle (-), the γ-secretase inhibitor DAPT (10 µM), the β-secretase BACE-1 inhibitor ELN582302 (Bi, 100 µM), the ADAM10 inhibitor GI254023X (GI, 10 µM), the general metalloprotease inhibitor GM6001 (GM, 10 µM), and the ADAM10/17 dual inhibitor GW280264X (GW, 10 µM). Cell lysates were probed by WB with anti-HA antibodies to detect FL-PLA2R1 and Cter-PLA2R1.

Supplementary Figure S6: Pro-inflammatory cytokines increase the secretion of Shed-PLA2R1 in HEK293 cells. Expression of hPLA2R1 was induced in T-REx™-293 cells by tetracycline treatment (1 µg/ml) for 48 h. Medium was changed and various concentrations (5 ng/ml, 10 ng/ml, 20 ng/ml) of the proinflammatory cytokines IL-6 (panel A), TNF-α (panel B) and IFN-γ (panel C) were added for 1 and 3 hours. Levels of Shed-PLA2R1 in cell medium was analyzed by WB. Histograms show the quantification of Shed-PLA2R1 from the WB.

Supplementary Figure S7: Quantification of circulating soluble mPLA2R1 in mouse serum by Time-Resolved FluoroImmunoAssay (TRFIA). A, Standard curve with recombinant purified soluble mPLA2R1 generated by plotting fluorescence counts over the concentration of soluble mPLA2R1 from 0 to 1,000 ng (Log scale). B, Concentration of circulating soluble mPLA2R1 in mouse serum from wild-type (mPLA2R1-WT) or knock-out (mPLA2R1-KO, *Pla2r1*$^{-/-}$) mice measured by TRFIA (n=6).

Table S1: Deletion mutants of human PLA2R1 used in this study. The Full-Length human PLA2R1 (FL-hPLA2R1) construct is based on the UniProt Q13018 sequence with the SNPs M292V and H300D. All deletion mutants are derived from this construct, and the amino acid sequence is as follows: each mutant harbors the signal peptide (SP) of hPLA2R1 ("^{1}MLLSPSLLLLLLLGAPRGCA^{20}") and the short N-terminal linker (Nter: "^{21}EGVAAALTPERLLEW^{35}") that precedes the Cysteine-rich domain (CysR) of hPLA2R1, and thus have the same N-terminal amino acid sequence (from M1 to W35). For some constructs, a 3xFlag tag ("DYKDHDGDYKDHDIDYKDDDDK") was inserted after this 35 amino acid N-terminal sequence. It is followed by the hPLA2R1 domains listed in the table below, where "Start" and "End" correspond to the amino acid numbering used in the UniProt reference sequence Q13018. All constructs comprise the intracellular region (Cter) and end at the C-terminal amino acid Q1463. All but one construct have a C-terminal HA tag ("YPYDVPDYA") after the Cter region. Some constructs have additional mutations, with targeted deletion of the entire juxtamembrane stalk (1383-1397), or part of it, or single point mutations at Proline 1390, upstream of the cleavage site of human PLA2R1 that was identified in non-stimulated conditions (Figure 2).

Construct name	Tags and domain borders	SP & Nter	3xFlag	Comprised Domains (AA) Start	End	HA	Additional mutation	Vector
Deletion mutants of hPLA2R1 (to study the structural determinants of hPLA2R1 cleavage)								
Full-Length hPLA2R1	CysR-Cter-HA	1-35	-	36	1463	Yes		pLPCX
ΔC	FNII-Cter-HA	1-35	-	165	1463	Yes		pLPCX
ΔF	CTLD1-Cter-HA	1-35	-	223	1463	Yes		pLPCX
Δ1	CTLD2-Cter-HA	1-35	-	357	1463	Yes		pLPCX
Δ2	CTLD3-Cter-HA	1-35	-	504	1463	Yes		pLPCX
Δ3	CTLD4-Cter-HA	1-35	-	660	1463	Yes		pLPCX
Δ4	CTLD5-Cter-HA	1-35	-	805	1463	Yes		pLPCX
Δ5	CTLD6-Cter-HA	1-35	-	947	1463	Yes		pLPCX
Δ6	CTLD7-Cter-HA	1-35	-	1105	1463	Yes		pLPCX
Δ7	CTLD8-Cter-HA	1-35	-	1235	1463	Yes		pLPCX
Δ7ΔShed	CTLD8-Cter-HA	1-35	-	1235	1463	Yes	Deletion 1383-1397	pcDNA3.1Zeo(-)
Construct for the inducible expression of hPLA2R1 in T-REx™ cells in pcDNA4/TO								
Full Length hPLA2R1		1-35	-	36	1463	Yes		pcDNA4/TO
Additional constructs with N-ter 3xFlag tag (Detection of shed fragment in cell medium)								
Full-Length hPLA2R1	CysR-Cter-HA	1-35	Yes	36	1463	Yes		pcDNA3.1Zeo(-)
PLA2R1ΔShed	CysR-Cter-HA	1-35	Yes	36	1463	Yes	Deletion 1383-1397	pcDNA3.1Zeo(-)
Δ7	3xFlag-CTLD8-Cter-HA	1-35	Yes	1235	1463	Yes		pcDNA3.1Zeo(-)
Δ7ΔShed	3xFlag-CTLD8-Cter-HA	1-35	Yes	1235	1463	Yes	Deletion 1383-1397	pcDNA3.1Zeo(-)
Additional deletion mutants of the Juxtamembrane Stalk								
Δ7-Δ1388-1393	3xFlag-CTLD8-Cter-HA	1-35	Yes	1235	1463	Yes	Deletion 1388-1393	pcDNA3.1Zeo(-)
Δ7-Δ1391-1393	3xFlag-CTLD8-Cter-HA	1-35	Yes	1235	1463	Yes	Deletion 1391-1393	pcDNA3.1Zeo(-)
Δ7-Δ1388-1390	3xFlag-CTLD8-Cter-HA	1-35	Yes	1235	1463	Yes	Deletion 1388-1390	pcDNA3.1Zeo(-)
Δ7-P1390D	3xFlag-CTLD8-Cter-HA	1-35	Yes	1235	1463	Yes	P1390D	pcDNA3.1Zeo(-)
Δ7-P1390G	3xFlag-CTLD8-Cter-HA	1-35	Yes	1235	1463	Yes	P1390G	pcDNA3.1Zeo(-)
Δ7-P1390A	3xFlag-CTLD8-Cter-HA	1-35	Yes	1235	1463	Yes	P1390A	pcDNA3.1Zeo(-)
